# From Monomers to Aggregates: The Influence of Redox State and Structure on the First Excited States of Eumelanin and Pheomelanin

**DOI:** 10.3390/ijms27135886

**Published:** 2026-06-30

**Authors:** Joanna Waresiak, Filip Sagan, Mariusz Paweł Mitoraj, Tadeusz Sarna

**Affiliations:** 1 Department of Biophysics, Faculty of Biochemistry, Biophysics and Biotechnology, Jagiellonian University, Gronostajowa 7, 30-387 Krakow, Poland; joanna.waresiak@doctoral.uj.edu.pl (J.W.); tadeusz.sarna@uj.edu.pl (T.S.); 2 Doctoral School of Exact and Natural Sciences, Jagiellonian University, Prof St. Lojasiewicza 11, 30-348 Krakow, Poland; 3 Department of Computational Methods in Chemistry, Faculty of Chemistry, Jagiellonian University, Gronostajowa 2, 30-387 Krakow, Poland; mitoraj@chemia.uj.edu.pl

**Keywords:** eumelanin, pheomelanin, ETS-NOCV, excited states, triplets, DHI, DHICA, benzothiazines, benzothiazoles

## Abstract

Melanin pigments protect human tissues from ultraviolet and visible radiation, yet their phototoxic potential increases with oxidative degradation. This computational study investigates how the oxidation state influences the first excited states of eu- and pheomelanin using molecular models of varying complexity (monomers to tetramers, both covalently and non-covalently bonded). First, vertical and adiabatic electronic transitions were computed, and supramolecular interactions were characterized with the ETS-NOCV method. In eumelanin, oxidation drastically lowers the first triplet-state (T_1_) energies (from above 230 kJ/mol) to levels comparable to retinal carotenoids (≤66 kJ/mol), emphasizing its role in triplet quenching rather than singlet oxygen generation. Pheomelanin showed greater heterogeneity in the values of the first triplet state, staying mostly above the eumelanin T_1_ energies. However, selected pheomelanin structures also exhibited relatively low triplet energies, particularly oxidized benzothiazole (BZox) and trichochromes, and although their T_1_ energetics remained higher than those calculated for oxidized eumelanin, they were still sufficiently low to suggest a potential ability to quench singlet oxygen. Furthermore, supramolecular analysis reveals that eumelanin aggregates are moderately stabilized by both π-π stacking and hydrogen bonding, whereas pheomelanin aggregates are dominated by dense hydrogen-bond networks.

## 1. Introduction

Melanins are heterogeneous biopolymers with unique physicochemical properties that facilitate their role as efficient photoprotective agents in pigmented tissues exposed to solar radiation [1,2]. The main function of melanin is to act as a broadband optical filter which limits the penetration of ultraviolet and short-wavelength visible radiation into tissues, thereby protecting cells from direct photodamage and from secondary photochemical effects related to generation of reactive oxygen species (ROS) leading to oxidative stress [3,4]. The protective function of melanin is also due to distinct redox properties [5], and the ability to quench excited states of different photosensitizers [6,7] and to chelate transition metals [8,9]. However, these protective properties of melanin may diminish with time. In the skin, melanin is continuously synthesized and degraded, allowing ongoing compensation for changes [10,11]. In contrast, melanin in the retinal pigment epithelium (RPE) is not renewed after embryonic development and undergoes cumulative photo-oxidative degradation throughout life [6,12]. These changes are accompanied by a reduction in melanin nanoaggregates, exposure to reactive chemical groups, modification of redox properties, and an increase in photochemical activity [13,14]. As a result, the pigment, undergoing age-related changes, may not only lose its ability to suppress oxidative stress but become a pro-oxidative agent by generating higher fluxes of ROS, including singlet oxygen, especially if the T_1_ energy level of such melanin shifts above the excitation threshold of ^3^O_2_.

Melanin deactivates electronically excited states predominantly via ultrafast nonradiative pathways, thereby limiting the population of long-lived excited states [15]. Due to the short lifetime of the excited state (S_1_) and the low yield of intersystem crossing to the first triplet state (T_1_), the pigment minimizes the risk of energy transfer to molecular oxygen and the formation of singlet oxygen. Nevertheless, the existence of triplet states in melanin has been experimentally demonstrated, despite their small population and transient nature [16]. Detailed characterization of specific photophysical and photochemical processes in melanin is challenging, due to unfavorable optical properties of the pigment, its intrinsic heterogeneity, aggregation, and ultrafast nonradiative relaxation that leaves only low, transient excited-state populations. Therefore, an experimental approach based on direct and selective detection of singlet oxygen, photogenerated by melanin, enables indirect characterization of the triplet states of the pigment.

Singlet oxygen is most commonly generated when the triplet-state energy of a sensitizer is sufficiently high to promote the transition of oxygen from its triplet ground state to the excited singlet delta state, i.e., about 95 kJ/mol, which can be monitored via the characteristic near-infrared phosphorescence emission of ^1^O_2_ at ~1270 nm. Notably, the effective ^1^O_2_ decay rate constant includes radiative, nonradiative, and reactive contributions. The radiative component corresponds to ^1^O_2_ phosphorescence near 1270 nm. The nonradiative component reflects physical quenching through collisions and energy dissipation without chemical transformation. The reactive component describes chemical consumption of ^1^O_2_ through oxidation reactions with available substrates. Nonradiative deactivation is governed mainly by diffusion-controlled bimolecular collisions. It is determined by three types of interactions: physical interactions with solute molecules, charge-transfer-type interactions, and interactions with solvent molecules. The first contribution corresponds to energy transfer from electronically excited ^1^O_2_ to the quencher, such that the quencher is promoted from its ground state to an excited state, typically a triplet, upon accepting the excitation energy from ^1^O_2_. This pathway is feasible only for quenchers whose triplet state lies below the energy of excited ^1^O_2_, ensuring an energetically allowed transfer. The charge-transfer-type rate constant describes a pathway in which deactivation of ^1^O_2_ is preceded by formation of a short-lived charge-transfer complex between ^1^O_2_ and a solute molecule present in solution. It is worth noting that melanins exhibit low quantum yields of ^1^O_2_ generation on the order of 10^−4^ to 10^−3^, but upon photodegradation or chemical degradation this efficiency may increase into the 10^−3^ to 10^−2^ range [14,17]; for comparison, the ^1^O_2_ quantum yield of methylene blue is about 0.52 [18]. Notably, lutein quenches ^1^O_2_ about two orders of magnitude more efficiently than DOPA-based melanin [19], whereas DOPA-based melanin quenches ^1^O_2_ about three orders of magnitude more efficiently than cholesterol [20]. Pheomelanin is typically a slightly less effective quencher of ^1^O_2_ than eumelanin [14,17]. Therefore, one of the approaches to better understand the behavior of melanin involves computational methods, especially those based on quantum chemistry, using representative melanin structures in various oxidation states [21,22].

**Figure 1:**
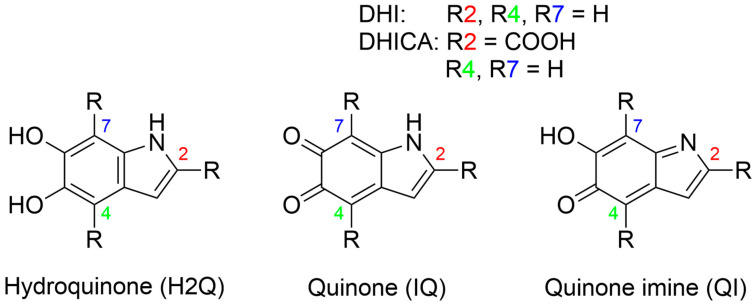
Schematic presentation of eumelanin DHI/DHICA subunits, with substituent/polymerization sites (positions 2, 4, 7) marked.

Most computational studies have focused on eumelanin models. Previous investigations demonstrated that eumelanin aggregation and interactions strongly depended on its molecular building blocks and charge state. DHI units readily form compact π-π stacks with interlayer distances of about 3.5 Å [23], whereas DHICA exhibits charge-dependent behavior: neutral forms aggregate and can entrap water molecules within the clusters, while deprotonated DHICA at physiological pH shows increased solubility, counterion binding, and reduced stacking propensity [24]. Another line of research addressed eumelanin–drug interactions, revealing that both DHI- and DHICA (Figure 1)-based eumelanin undergo conformational changes upon interaction with small molecules, leading to the formation of cavities and allosteric binding sites [25]. Yet another area of computational research has been devoted to elucidation of the continuous absorption spectrum. TD-DFT and MP2 studies have shown that the reduced H2Q species do not absorb in the visible region, whereas the oxidized IQ/QI species produce distinct UV and visible bands. In particular, DHI monomers and dimers in the oxidized forms exhibit intense absorption in the visible region and higher extinction coefficients compared to DHICA, whose carboxyl group limits planarity and shifts absorption toward the UV [26]. However, these studies were largely limited to singlet states, without addressing the triplet states that play a crucial role in photochemical reactions, particularly focusing on the generation/quenching of singlet oxygen in melanin-containing systems. It has been pointed out that optical absorption and emission of eumelanin can be explained by a simple heterogeneous mixture of oligomeric species, in which chemical and physical disorder as well as inhomogeneous line broadening play a substantial role [27].

To this end, we analyze herein for the first time how the type of melanin pigment, its oxidation state, and molecular aggregation influence the excited-state properties, with particular emphasis on the position and function of the triplet state. Using DFT/B3LYP/ETS-NOCV calculations for structural and bonding analysis, and DFT/CAM-B3LYP for photochemistry, we systematically investigate monomers, dimers, trimers, and tetramers of basic melanin building blocks, considering both covalently bonded oligomers and non-covalent molecular assemblies. Physical factors determining formation of various aggregates differing in size are also delineated and discussed. Our goal is to deepen the understanding of the already challenging photochemistry of melanin by discussion of the balance between the singlet oxygen generation and quenching capabilities of increasing-size aggregates. This work fits well into recent frontier and difficult research on supramolecular photochemistry where aggregation and/or other physical interactions are decisive for photoactivity [28,29,30,31,32].

## 2. Results and Discussion

### 2.1. Eumelanin Building Units

#### 2.1.1. DHI- and DHICA-Based Monomers

Although eumelanin and pheomelanin share a common biosynthetic precursor, L-3,4-dihydroxyphenylalanine (L-DOPA), their synthetic pathways diverge at a defined stage of melanogenesis [10,33,34,35]. A pivotal branching point occurs upon the reaction with cysteine: its presence facilitates the formation of pheomelanin, whereas its absence promotes the synthesis of eumelanin [21,36,37]. This biosynthetic bifurcation is reflected at the structural level of the respective pigment monomers. In the case of eumelanin, monomers are based on the 5,6-dihydroxyindole skeleton, containing a hydrogen atom in position 2 for DHI, and a carboxylic group for DHICA (Figure 1). Possible oxidation of the hydroxylic and amine groups forming the quinone (IQ) and quinone imine (QI) variants leads to six basic monomeric units, denoted with both abbreviations in further parts of this work (e.g., DHI-H2Q is a hydroquinone variant of the DHI molecule) [38]. For example, DHI can occur as DHI-H2Q (reduced hydroquinone form), DHI-IQ (oxidized quinone form), or DHI-QI (its quinone–imine tautomer). All of them exhibit a planar optimal geometry, both in the gas phase and in water.

**Figure 2:**
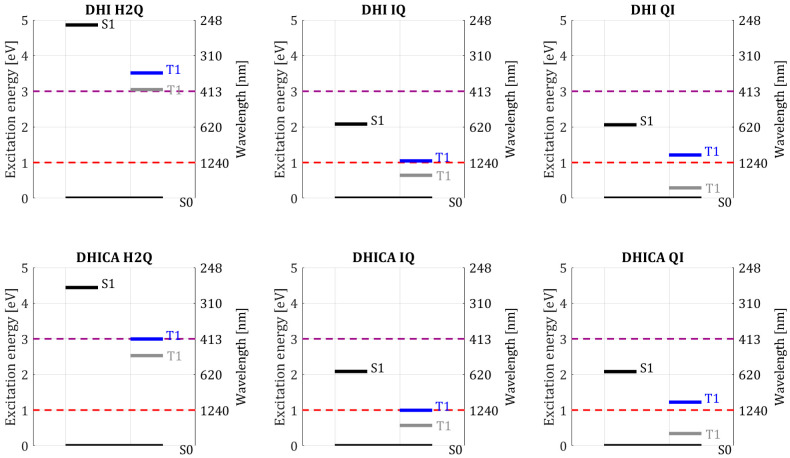
Location of the first triplet (T_1_) and singlet (S_1_) states for eumelanin subunits DHI and DHICA in other redox states (H2Q, IQ, QI). The values determined as a vertical transition are presented (S_1_ in black, T_1_ in blue), while adiabatic T_1_ energies are shown in grey. Additionally, the red line is the boundary of singlet oxygen phosphorescence, and the purple line is the boundary associated with the light that is able to reach the retina (below this line) [42].

Analysis of the first excited states of eumelanin monomers revealed two distinct triplet energy fractions: high-energy triplets associated with fully reduced subunits (H2Q) and low-energy triplets corresponding to oxidized forms (IQ and QI) (Figure 2). The DHI-H2Q models exhibited higher excitation energies for both the S_0_ → S_1_ and S_0_ → T_1_ transitions compared to their DHICA H2Q counterparts. The calculated S_0_ → T_1_ vertical transition energy for the oxidized structures was approximately 100 kJ/mol. The concomitant lowering of the S_1_ → T_1_ transition relative to H2Q forms facilitates triplet formation. Reduced monomers position the S_1_ state predominantly in the UV range, whereas oxidized species position S_1_ at lower energies (red-shifted), toward longer wavelengths and into the visible region. Despite only minor geometric changes between the ground state (S_0_) and the first excited singlet state (S_1_) (Figure S1), adiabatic transition calculations revealed further lowering of excitation energies compared to vertical excitations (Table 1). Notably, QI forms exhibited significantly lower excited-state energies than IQ, indicating a greater contribution of QI structures to the formation of low-energy triplet states. These energy levels fall within the vibrational range of C=O and N–H/O–H groups [39]. Nonetheless, the low triplet energies likely render them incapable of initiating the conversion of ground-state triplet oxygen to its singlet state; instead, these values suggest energetic compatibility with singlet oxygen quenching. Nevertheless, the possibility of singlet oxygen generation, albeit with very low efficiency, cannot be excluded. Considering that melanin is synthesized and stored in melanosomes, which are acidic at early stages but alkalinize toward near-neutral pH upon maturation [40], and that the pH of synthetic-melanin suspensions can decrease during aerobic photodegradation [14], the protonation state of the building blocks can change. Given that the carboxylic acid group of DHICA has a pKa of approximately 4.2, at physiological 7.4 pH the molecule predominantly exists in its deprotonated form. The resulting –COO^−^ moiety imparts a negative charge, influencing solubility and intermolecular interactions [41]. To account for this, we also investigated the excited-state properties of the deprotonated DHICA model. This charged species showed slightly higher energies for the S_1_ → S_0_ transition in the case of the reduced H2Q form by 17 kJ/mol and for the T_1_ →S_0_ transition by 21 kJ/mol compared to the fully protonated form. In the case of the oxidized forms, this difference was not greater than 13 kJ/mol (Table 1). While the elevated S_0_ → S_1_ energy suggests decreased photo-responsiveness, the increase in T_1_ → S_0_ energy may enhance the potential for singlet oxygen generation.

Nevertheless, the energy differences were modest, and subsequent analyses were focused on the fully protonated systems. It is also worth noting that deprotonated DHICA exhibits distinct aggregation behavior and stronger surface interactions compared to its neutral counterpart [24]. As reported by Soltani et al., in the case of uncharged DHICA-based systems, spontaneous aggregation in water was observed, although the twisted molecular geometry limited regular planar stacking and allowed water molecules to remain within the aggregates. In contrast, fully deprotonated DHICA systems, corresponding to physiological pH, showed increased solubility because the negatively charged carboxyl groups favored interactions with water and K^+^ counterions. In mixed systems, charged DHICA units were located mainly at the surface of aggregates formed by uncharged units, indicating that protonation state may influence aggregate morphology, hydration, surface charge and chromophore exposure. For comparison, DHI-based systems were described using a tetrameric planar model, which favored regular π–π stacking and reduced conformational flexibility; unlike DHICA aggregates, these DHI stacks did not contain water molecules between the aggregated layers [23].

#### 2.1.2. DHI- and DHICA-Based Dimers

**Figure 3:**
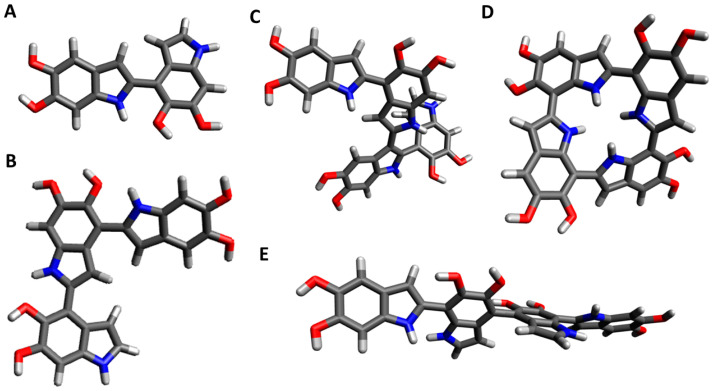
Spatial structure of DHI structures: 24 DHI-H2Q dimer (**A**), 42-42 trimer (**B**), 24-23-24 tetramer (**C**), 27-23-27 tetramer (**D**) and 27-44-72 tetramer (**E**).

In the next phase of the study, we extended our investigation to dimeric eumelanin structures composed of DHI and DHICA subunits. The monomers under study can form both covalently and non-covalently bonded dimers. In the first group, the monomers can link in the positions denoted 2, 4 and 7 (Figure 1). In the text, they will be described by the linkage position for both monomers, followed by the monomer abbreviation; e.g., 24-DHI-H2Q describes the homodimer of DHI-H2Q units connected by the 2 and 4 positions to each other. It is worth noting that the optimal structure of the DHI dimers is planar, whereas longer chains cannot adopt a planar geometry due to geometric constraints (Figure 1 and Figure 3).

**Figure 4:**
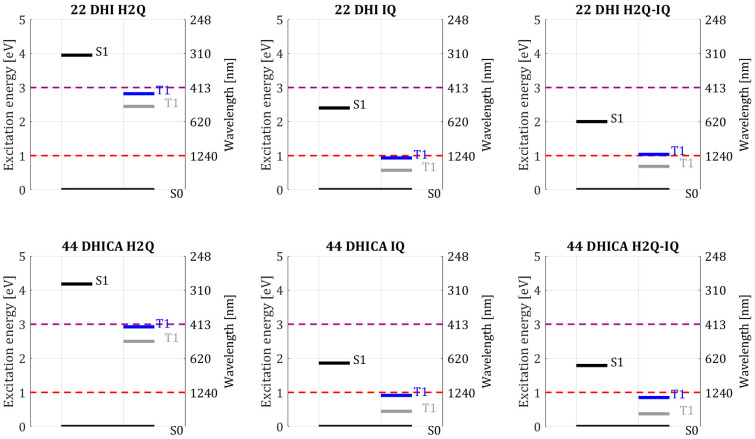
Localization of the first triplet (T_1_) and singlet (S_1_) states for the eumelanin dimers 22-DHI H2Q, 22-DHI IQ, 22-DHI H2Q-IQ, 44-DHICA H2Q, 44-DHICA IQ, and 44-DHICA H2Q-IQ. The values determined as a vertical transition are presented (S_1_ in black, T_1_ in blue), while adiabatic T_1_ energies are shown in grey. Additionally, the red line is the limit of singlet oxygen phosphorescence, and the purple line is the limit associated with light that can reach the retina (below this line).

In the case of covalently bonded eumelanin dimers, their general excited-state energetics are comparable with the monomeric models—they cluster into two main fractions corresponding to reduced and oxidized forms, as seen in both singlet and triplet states. Dimerization, however, makes DHI and DHICA much more similar to each other in terms of their S_0_ → S_1_ and S_0_ → T_1_ transition energetics. The first triplet-state energy in reduced (H2Q) DHI dimers ranges from 288 kJ/mol in the case of 27-DHI-H2Q to 306 kJ/mol for 24-DHI-H2Q (Figure 4 and Table 1), which is markedly lower than 339 kJ/mol in the case of monomers. This can be potentially attributed to increased electron delocalization in dimers, stabilizing the triplet state (Figure S2). This lowering brings DHI closer to DHICA triplet energetics, with a transition energy of 282 kJ/mol for 44-DHICA-H2Q, compared to 289 kJ/mol for a monomer. Oxidized IQ and QI forms follow suit, ranging between 71 and 111 kJ/mol, without any clear trends differentiating DHI- and DHICA-based systems, but with slightly lower energies for quinone (QI) variants (Table 1, Figure 4). Mixed redox dimers, in which one monomer was reduced (H2Q) and the other oxidized (IQ or QI), were also evaluated, including both DHI- and DHICA-based systems. Their triplet energies were comparable to those of fully oxidized dimers and significantly lower than those of fully reduced forms, as reflected by the localization of the triplet excitation on an oxidized monomer (Figure S2). Thus, even partial oxidation within a dimer seems to be sufficient to markedly lower the triplet energy value, highlighting its sensitivity to the oxidation state.

#### 2.1.3. Non-Covalently Bonded Dimers

**Figure 5:**
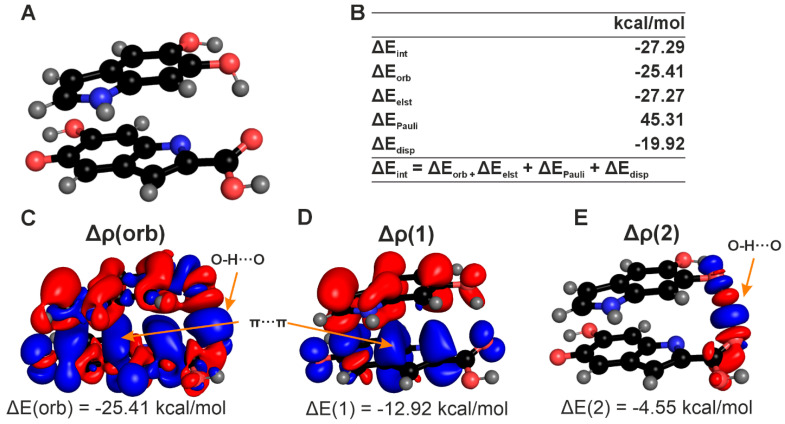
ETS-NOCV analysis of the most stable DHI/DHICA dimer. Optimized structure of the complex (**A**); ETS-NOCV energy decomposition (**B**); total orbital deformation density (**C**); NOCV deformation density contribution associated mainly with π–π stacking stabilization (**D**); NOCV deformation density contribution associated mainly with hydrogen-bond stabilization (**E**); Blue and red indicate electron density accumulation and depletion, respectively.

Possible global minima of non-covalently bonded dimers were explored for all monomer combinations of DHI and DHICA units. Two dominant binding motifs are observed in the studied complexes: H-bond (O---HO)-driven dimer formation, and stacking aggregation facilitated by π-π interactions. Mixed forms were also observed; in fact, the most stable complex incorporates both stacking and hydrogen bonding (Figure 5). The interaction energies of these complexes range from −10 to −27 kcal/mol, and the most stable ones contain one or two hydrogen bonds. On average, systems containing DHICA are more stable by ca. 2 kcal/mol (Table S1).

The most stable non-covalently bonded dimer found (ΔE_int_ = −27.3 kcal/mol) consists of DHICA-QI and DHI-H2Q molecules. The energy decomposition analysis shows that stabilizing orbital overlap, electrostatic interaction and dispersion are comparable in magnitude, accounting for 35%, 37% and 27% of total stabilization, respectively, highlighting the synergistic role of stacking and hydrogen bonding in complex formations. Our results are consistent with experimental results indicating that eumelanin forms layered aggregates in which π–π interactions occur, often described by a stacked-oligomer model with small “tiles” (4–6 indole units) arranged at an interlayer spacing of ~0.34 nm [43,44,45,46]. This has also been corroborated by the theoretical simulations reported by Soltani et al. [23,24], whose studies consistently demonstrate the preference of eumelanin fragments to organize into stacked architectures.

As far as their photochemistry is concerned, the reduced/oxidized-form distinction between the species is continued—dimers consisting solely of H2Q forms are characterized by the first vertical triplet energies at the level of ca. 290 kJ/mol, characteristic for monomers. Mixed (in terms of the oxidation state between monomers) dimers in general exhibit the character of the oxidized species, with a T_1_ vertical state at ~100 kJ/mol (Figure S13). This strongly suggests that intermolecular non-covalent interactions modify the photochemistry of monomers only to a minor degree. However, the presence of explicitly treated water molecules or further melanin subunits may change this behavior. In particular, explicit hydration may compete with intermonomer hydrogen bonds and modify the balance between stacked and hydrogen-bonded arrangements. This is consistent with experimental evidence showing that solvent, pH and oligomer size influence excited-state deactivation in DHICA-based eumelanin building blocks [47].

#### 2.1.4. Larger Covalently Bonded Systems

To determine whether the established trends hold in larger eumelanin structures, we selected several DHI and DHICA trimers for our study (for details, see the section “DHI and DHICA-based trimers and tetramers” in ESI). As was the case in dimeric structures, triplet energetics in DHI tri- and tetramers became much more similar to DHICA multimers when compared to the monomers, probably owing to the extended delocalization of triplet excitation within the planar aromatic ring. This is also the case for the tetramers, where completely planar structures are no longer possible due to geometric constraints, but local (i.e., dimer/trimer-like) conjugated fragments exist (Table 1). Likewise, the clear distinction between oxidized (QI/IQ) and reduced (H2Q) systems is continued, with the latter exhibiting much lower-lying T_1_ states.

#### 2.1.5. Larger Non-Covalently Bonded Systems

**Figure 6:**
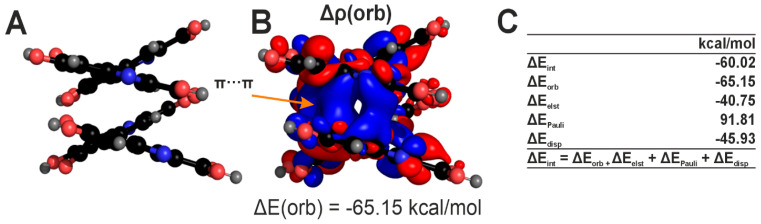
ETS-NOCV analysis of the most stable dimer of DHICA dimers, Optimized structure of the stacked complex (**A**); total orbital deformation density, Δρ(orb) (**B**); ETS-NOCV energy decomposition of the interaction between the dimeric fragments (**C**); Blue and red indicate electron density accumulation and depletion, respectively.

As an example of larger structures of melanin, non-covalently bound dimers of DHICA homodimeric subunits of different oxidation states and selected DHI dimers were considered. DHI dimers continued to stack favorably, and the most stable minimum found for each system showcased efficient π-π interactions. Oxidized variants of DHI continued to exhibit far lower triplet energies than the reduced ones (Figure S10). In the case of DHICA, dimers with a high number of hydrogen bonds (up to four) tend to be more stabilized, but the stacking motif is still present in the most stable structures. For example, the most stable one is bound primarily by π-π stacking (Figure 6), with orbital interaction energy being the most important stabilizing factor (43%), followed by dispersion (30%) and electrostatics (27%). Notably, enlargement of interacting systems (interacting dimers compared to monomers) led to a cooperative increase in stabilization—the most stable DHICA homodimer found, stabilized by a reciprocal double hydrogen bond, exhibited ΔE_int_ = −26.6 kcal/mol. The other most stable minima combined both bonding modes.

### 2.2. Pheomelanin Building Units

#### 2.2.1. BT- and BZ-Based Monomers

**Figure 7:**
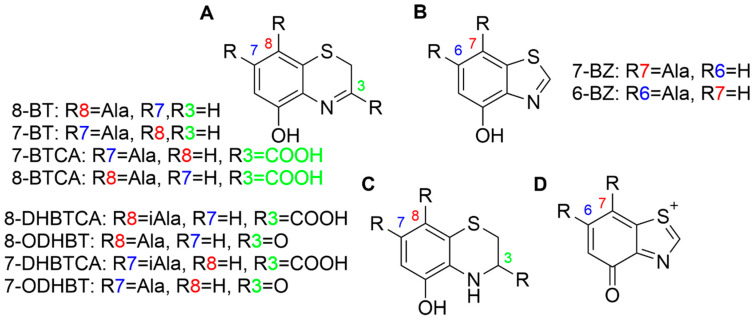
Schematic presentation of pheomelanin BT and BTCA subunits (**A**), their oxidized counterparts, DHBTCA and ODHBT (**C**), and BZ (**B**) and its oxidized form, BZox (**D**) [21].

Pheomelanin monomers are benzothiazole and benzothiazine derivatives: (2-amino-2-carboxyethyl)-4-hydroxybenzothiazole (BZ) and (2-amino-2-carboxyethyl)-4-hydrobenzothiazine (BT). Depending on the position of their 2-amino-2-carboxyethyl moiety, they will be referenced as 6- or 7-BZ and 7- or 8-BT (Figure 7). BTCA is BT substituted in the 3- position by a carboxylic group, and DHBTCA is its oxidized form, with amine in place of imine. There is also ODHBT, a 3-oxo derivative of oxidized BT. Although all listed isomers were considered, the results obtained were very similar, and only representative values are presented in this work.

**Figure 8:**
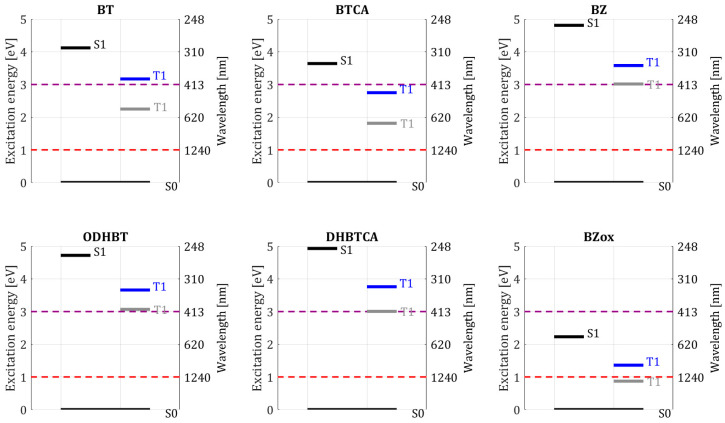
Location of the first triplet (T_1_) and singlet (S_1_) states for pheomelanin monomers in different redox states. The values determined as a vertical transition are presented (S_1_ in black, T_1_ in blue), while adiabatic T_1_ energies are shown in grey. Results for 7-BT, 7-BZ etc. isomers are shown. Additionally, the red line is the boundary of singlet oxygen phosphorescence, and the purple line is the boundary associated with the light that is able to reach the retina (below this line).

The only monomer with a fully planar condensed ring structure is BZ. In BT and its derivatives, six-membered sulfur rings are not aromatic, and hence, not planar. Pheomelanin is widely recognized as the more photoreactive form of melanin, often associated with increased photosensitizing potential [17,48]. This, however, is not reflected clearly in the T_1_ energetics of the monomers—both BZ and, to a lesser extent, BT are rather similar to eumelanin, with 345 kJ/mol and 306 kJ/mol, respectively (compared to 339 kJ/mol for DHI and 289 kJ/mol for DHICA) (Figure 8). The model containing the carboxylic group BTCA exhibits a noticeably lower-lying T_1_ state: 265 kJ/mol. Even more striking is the fact that the oxidized BT versions—DHBTCA and ODHBT—have their triplets even higher, at 362 kJ/mol and 353 kJ/mol, respectively. Conversely, the model of oxidized BZ—BZox—stands out with its very low S_0_-→T_1_ transition energy of 132 kJ/mol. The T_1_ level is low enough to be populated by visible light excitation; in contrast, the remaining pheomelanin subunits have markedly higher T_1_ energies, making them poorer triplet quenchers under visible irradiation and under sufficiently energetic excitation potentially capable of sensitizing singlet oxygen. Importantly, however, the T_1_ energy of oxidized BZ remains higher than the values typically reported for oxidized eumelanins.

Notably, the excited-state geometries of pheomelanin monomers showed greater structural reorganization than those of their eumelanin counterparts (Figures S1 and S13). In several cases, optimization of triplet states often converged to different local minima, reflecting a complex excited-state conformational landscape. This reflects the increased structural flexibility of pheomelanin, which may improve accessibility to small molecules such as oxygen, potentially promoting singlet oxygen formation.

#### 2.2.2. BT- and BZ-Based Dimers

**Figure 9:**
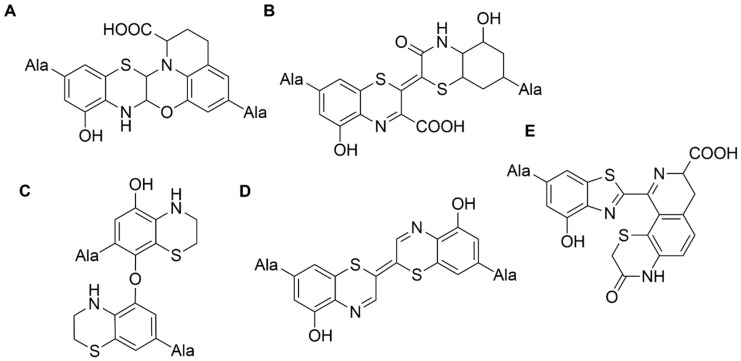
Covalent dimers as oxidation products of BT ((**A**): cyclo, (**C**): dimer) and BTCA ((**B**): trichochrome 1, (**D**): trichochrome 2, and (**E**): BT-TQ) [21].

**Figure 10:**
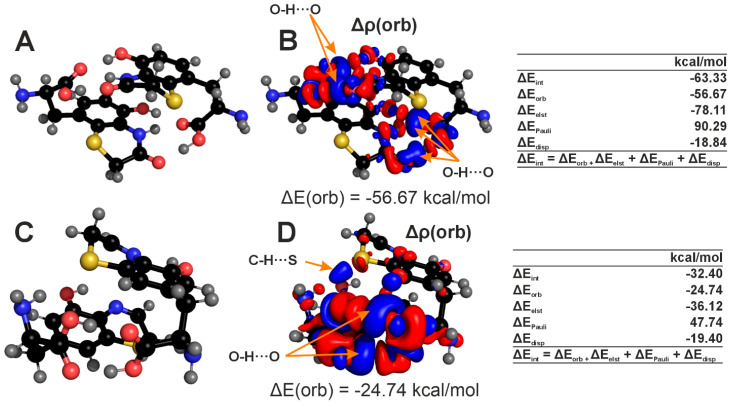
ETS-NOCV analysis of the most stable dimer of pheomelanin subunits, the homodimer of 8-ODHBT, exhibiting multiple hydrogen bonds (**A**,**B**), together with the least stable one, the 7-BT homodimer, showcasing two hydrogen bonds and two N-H---S interactions (**C**,**D**). Blue and red indicate electron density accumulation and depletion, respectively.

The oxidation of cysteinyldopa isomers leads to benzothiazine (BT) intermediates that undergo dimerization, cross-coupling, and/or ring contraction to benzothiazole (BZ) moieties through complex reactions. Given the structural diversity of pheomelanin monomers and their extensive modification during polymerization, we analyzed only a selected set of representative dimers. The examined set includes both well-known oxidation products of 1,4-benzothiazine (BT) and its carboxylated derivatives (BTCA), as well as more complex structures found in mature pheomelanin pigment. Specifically, we included a cycloaddition product (Figure 9A), a classical C–O BT dimer (Figure 9C), and two compounds classified as trichochromes: trichochrome 1 (Figure 9B) and trichochrome 2 (Figure 9D). Trichochromes are natural components of pheomelanin, as is BT-TQ (Figure 9E), a compound containing benzothiazole and isoquinoline rings, also identified in red hair and feathers [21,49,50]. In the case of non-covalently bound dimers, we used the regular (i.e., unmodified) monomers for complex creation. All possible pairings of monomers were considered. Contrary to the eumelanin subunits, pheomelanin monomers tend to interact by means of hydrogen bonding more than stacking. All minima have at least two intermolecular hydrogen bonds, and the most stable showcase 4–6 of them. Due to this fact, their stabilization is more pronounced, at between −30 and −60 kcal/mol. The most stable complex is the homodimer of 8-ODHBT, featuring six hydrogen bonds, which give rise to ΔE_int_ = −63 kcal/mol, jointly due to electrostatics (51%) and orbital overlap (37%), followed by dispersion (12%) (Figure 10). Interestingly, complexes of the non-oxidized forms of BT and BZ are stabilized less than the ones containing their oxidized forms (Figure 10C,D; Table S2, SI). This is consistent with the additional capabilities for hydrogen bonding provided by added carboxyl groups in oxidized species. Increasing the number of hydrogen-bond donors and acceptors also enhances solubility, which in turn allows for easier water access and, counterintuitively, may be one of the reasons for easier fragmentation of pheomelanin aggregates compared to eumelanin, despite the stronger interactions between the monomers [51,52].

**Figure 11:**
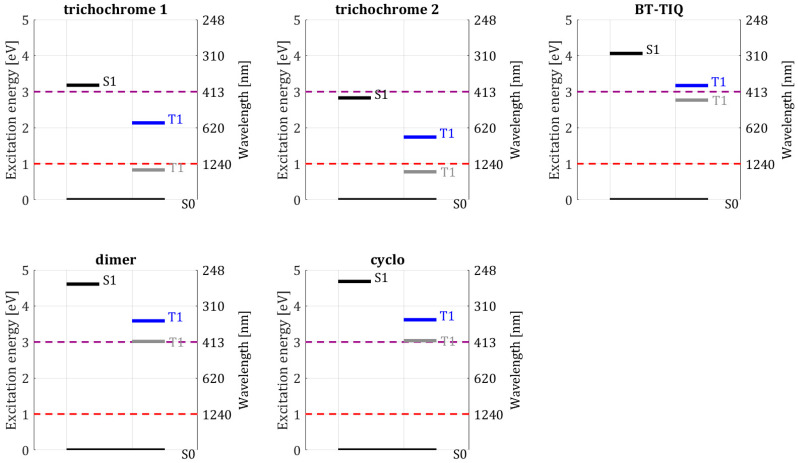
Location of the first triplet (T_1_) and singlet (S_1_) states for pheomelanin dimers in different redox states. The values determined as a vertical transition are presented (S_1_ in black, T_1_ in blue), while adiabatic T_1_ energies are shown in grey for BTCA oxidation products (t trichochromes 1–2 and BT-TIQ) and for BT oxidation products (dimer and cyclo). Additionally, the red line is the boundary of singlet oxygen phosphorescence, and the purple line is the boundary associated with the light that is able to reach the retina (below this line).

The photochemistry of pheomelanin dimers was explored using representative oxidation products of BTCA and BT units, including trichochromes 1 and 2, BT-TIQ, and dimeric derivatives (Figure 9). These structures correspond to key proposed photodegradation products and covalent rearrangement products reported in experimental studies. BT dimeric derivatives continue the trend set up by BT oxidation products with T_1_ energies around 345 kJ/mol (Table 1). This agrees well with the results of previous studies, which observed increased oxygen uptake and photoionization of pheomelanin after excitation at wavelengths between 338 and 323 nm (i.e., between 354 and 370 kJ/mol) [53,54]. Slightly lower triplet energy was identified in BT-TIQ, with T1 at 306 kJ/mol, featuring the lowest S_1_-→T_1_ energy gap among all studied models, suggesting a higher propensity for triplet-state formation (Figure 11). This is consistent with experimental studies, which identified BT-TIQ as one of the main photodegradation products of benzothiazine-based pheomelanin units and a compound capable of sensitizing the formation of singlet oxygen [55].

Trichochromes were the subject of in-depth experimental photothermal measurements as well as transient absorption spectroscopy studies, which concluded that more than 90% of the absorbed photon energy is dissipated as heat, while only ~15% leads to the formation of a long-lived intermediate excited state with an estimated energy of ~133 kJ/mol [54]. Our calculations resulted in similar lowering of T_1_ state energy—206 and 168 for trichochromes 1 and 2, respectively (interestingly, B3LYP calculations return 133 kJ/mol for trichochrome 2; Tables S4 and S5 in SI)—which confirms the physics of our adopted model. Additionally, considering the adiabatic approach, which resulted in T_1_ energy of ca. 80 kJ/mol for both trichochrome models, enabling quenching by singlet oxygen (Figure 11). Importantly, despite this low value, T_1_ values of the abovementioned pheomelanin systems are still higher than for oxidized forms of eumelanin.

#### 2.2.3. Larger Non-Covalently Bonded Systems

**Figure 12:**
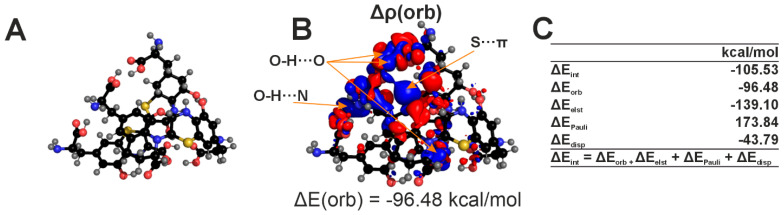
ETS-NOCV analysis of the most stable dimer of the large pheomelanin subunit dimers, exhibiting multiple hydrogen bonding. Optimized structure of the complex (**A**); total orbital deformation density (**B**); ETS-NOCV energy decomposition of the interaction between the fragments (**C**). Blue and red indicate electron density accumulation and depletion, respectively.

Due to the increasing complexity of the pheomelanin polymers, only non-covalently bonded adducts of dimeric subunits were studied. Similarly to DHICA dimers, all of them stacked, but due to their larger inherent flexibility all of them managed to form hydrogen bonds as well. The most stable stacks reached remarkable stabilization of c.a. −100 kcal/mol, mainly due to the large number of hydrogen bonds (the most stable complex exhibits eight connections, not counting intramolecular ones). Contrary to the eumelanin subunits, stacking is not a dominant factor in their aggregation, and this is evident in the energy components as well: for the most stable aggregate, the electrostatics is the most important (50%), followed by orbital interactions (35%), and dispersion is the least significant (15%) (Figure 12). This interaction profile markedly differs from that of eumelanin, where dispersion and π–π stacking play a more prominent role. These results are consistent with the X-ray diffraction data for pheomelanin, where a broad, diffuse maximum is observed at a position similar to eumelanin but with lower intensity. This suggests greater structural disorder in pheomelanin compared to eumelanin [46]. The difference may reflect not only the intrinsic polarity of the pheomelanin building blocks, but also the biological context of pigment assembly. Eumelanin is deposited onto a structured protein scaffold formed by PMEL17 fibrils, which organize the polymer into a fibrillar architecture and reduce the need for strong pigment–pigment interactions [56]. In this scaffolded environment, stacking interactions and dispersion forces may contribute substantially to supramolecular organization. In contrast, pheomelanin is synthesized in the absence of PMEL17, due to its repression by the Agouti signaling protein (ASP). As a result, pheomelanosomes exhibit irregular morphology and lack internal fibrillar templating. Pigment accumulates as granular deposits within multivesicular vesicles, without a defined template [57]. In such a disordered system, direct intermolecular interactions—particularly electrostatics and hydrogen bonding—become the dominant forces governing assembly. The photochemistry of these complexes largely follows the trends observed in their dimers—systems containing trichochrome dimers show vertical triplet excitation energies between 96 and 193 kJ/mol, whereas the rest exhibit T_1_ energies of ca. 289 kJ/mol (Figure S14).

### 2.3. Melanin Degradation Products, Retinal Carotenoids and Selected Reference Probes

**Figure 13:**
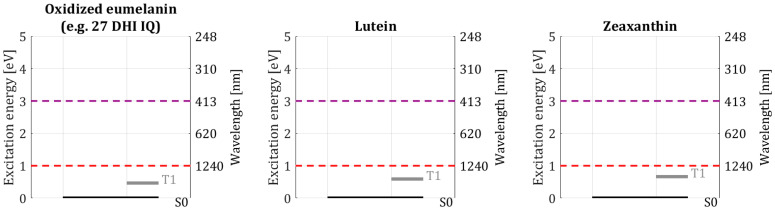
Location of the first triplet (T_1_) states for oxidized melanin (dimer 27-DHI IQ), lutein and zeaxanthin. The values determined as an adiabatic transition are presented (T_1_ shown in grey). Additionally, the red line is the boundary of singlet oxygen phosphorescence, and the purple line is the boundary associated with the light that is able to reach the retina (below this line).

Due to the observed very low values of the states, especially the triplet states of oxidized eumelanin, we attempted to determine how they relate to the triplet states of retinal carotenoids [58], which, as we know, exhibit some of the lowest triplet states occurring in nature. The obtained results suggest that the first excited triplet states have similar or even lower values of these states, which may help explain the mechanism of the reaction of quenching singlet oxygen by melanin (Figure 13). For lutein, the T_1_ value for the adiabatic transition was 57 kJ/mol, and for zeaxanthin it was 64 kJ/mol (Table 1). It is worth noting that under photosensitized oxidative stress, lutein and zeaxanthin can be converted into oxidation products, including 5,8-endoperoxides and aldehydic derivatives, which progressively depletes the pool of intact carotenoids and weakens their protective function [59]. Consequently, the system may become enriched in derivatives with altered photochemistry and an excited-state landscape distinct from that of the parent pigments. In this context, the exceptionally low T_1_ energies we obtained for oxidized eumelanin suggest that melanins may remain effective quenchers of triplet excitations of carotenoid oxidation products. In particular, if a given oxidative pathway disrupts the continuity of the conjugated π-system or yields shorter chromophoric fragments, shifts in T_1_ relative to the parent compounds are expected, which would favor energy-transfer channels to melanin [60]. Since carotenoids have many conjugated bonds, similar to oxidized melanin, this may slightly distort the accuracy of the results; the tendency for the energy of the lowest triplet to monotonically decrease with increasing conjugated bond chain length is retained [60]. Additionally, specific chemical degradation products of eumelanin (PDCA, PTCA, PTeCA) and pheomelanin (TDCA, TTCA), as well as the aminohydroxyphenylalanine 3-AHP and 4-AHP (collectively referred to as AHP), were characterized [61]. The first excited states estimated for these degradation products suggest that they all exhibit very high energies for the singlet excited state (S_1_), as well as similarly elevated values for the triplet state (T_1_). AHP derivatives exhibited the highest excited-state energies among all compounds analyzed. The lowest values (~250–260 kJ/mol) were observed for molecules containing benzothiazole fragments, intermediate energies were found for pyrrole-based markers derived from eumelanin, and the highest values (~310 kJ/mol) were associated with AHP-type structures.

These markers are expected to arise from oxidative depolymerization and fragmentation pathways and may therefore represent comparatively late-stage degradation products; nevertheless, they are routinely quantified in complex systems, which justifies their inclusion in the present energetic analysis [14]. The estimated excited-state energies indicate uniformly high T_1_ values in the visible-energy range, making the quenching of low-energy triplet excitations by these molecules unlikely on energetic grounds. Instead, upon photoexcitation they can populate a triplet excited state and behave as type II photosensitizers, enabling energy transfer to ground-state oxygen and, consequently, singlet oxygen generation, particularly under conditions of sufficient oxygen availability and limited contribution of competing deactivation pathways.

Melanin photoreactivity studies often involve the detection of singlet oxygen phosphorescence, generated by the photoexcited melanin. The yield of singlet oxygen photogeneration is determined by a comparative method, in which established photosensitizers—such as the positively charged methylene blue and the neutral riboflavin—are photoexcited under similar experimental conditions as melanin. Triplet-state quenchers like sorbic alcohol are used to probe excited triplet states of melanin [62,63]. To validate the reliability of our computational approach, we calculated the excited-state energies for these three reference compounds. For vertical transitions (Table 1), our calculated T_1_ energies are in good agreement with the experimental data, falling within the reported uncertainty ranges. Specifically, methylene blue, which has an experimentally reported T_1_ energy of approximately 142 kJ/mol [64,65], showed a calculated value of 138 kJ/mol for the vertical transition and 120 kJ/mol for the adiabatic transition. Riboflavin, with a known T_1_ energy in the range of 202–209 kJ/mol [65,66], yielded a calculated value of 227 kJ/mol for the vertical transition and 195 kJ/mol for the adiabatic transition. Sorbic alcohol, which is capable of quenching triplets above 250 kJ/mol [62], displayed 317 kJ/mol for the vertical transition and 226 kJ/mol for the adiabatic transition. The best agreement with the experimental data was obtained for methylene blue, where the calculated vertical T_1_ energy differed by only −4 kJ/mol from the reported value. For riboflavin, the vertical transition was overestimated by 18–25 kJ/mol, while the adiabatic result fell within 7–14 kJ/mol below the experimental range.

For classical water-soluble reference photosensitizers such as methylene blue, the singlet oxygen quantum yield can vary with sensitizer concentration. A pronounced decrease in the singlet oxygen quantum yield at higher concentrations has been attributed to self-quenching and molecular aggregation, which reduce the efficiency of energy transfer from the photosensitizer triplet state to molecular oxygen. This dependence highlights an interpretational pitfall, namely that apparent singlet oxygen generation efficiencies may reflect not only intrinsic photophysics but also aggregation-controlled accessibility and deactivation pathways. In melanin systems this issue is amplified because strong aggregation and limited chromophore exposure can suppress photochemical readouts and obscure the balance between singlet oxygen production and singlet oxygen quenching, which may shift as the aggregation state changes [67]. Importantly, melanin binds substantial amounts of iron [68,69], and such heavy atoms can enhance spin–orbit coupling via the heavy atom effect, thereby increasing the probability of intersystem crossing by promoting mixing between electronic states of different multiplicities. This effect becomes more pronounced when singlet and triplet vibronic levels are close in energy or partially overlap, which further facilitates the singlet to triplet transition [70].

In the case of sorbic alcohol, larger discrepancies were observed, with the vertical transition overestimated by ~67 kJ/mol and the adiabatic value underestimated by ~24 kJ/mol relative to the experimental threshold of 250 kJ/mol (though it should be noted that sorbic acid displays lower values of around 200 kJ/mol [63]). Notably, calculations performed at the B3LYP level provided a better overall match to experimental values. This is relevant because sorbic alcohol is commonly used as a triplet probe, quenching triplet excited states whose energies exceed the probe’s own T_1_ [62]. In heterogeneous systems, the efficiency of such quenching can depend not only on energetic alignment but also on diffusion and site accessibility, that is, on how frequently the probe can physically encounter a given chromophore. In this context, our results are particularly informative because they indicate pronounced differences in the interactions stabilizing eumelanin and pheomelanin aggregates: eumelanin aggregates are moderately stabilized by a combination of π–π stacking and hydrogen bonding, whereas pheomelanin aggregates are dominated by dense hydrogen-bond networks. This implies distinct microstructures and probe accessibility, and therefore coupling triplet probe measurements with further modeling of chemically modified melanins and systematic experimental validation should help disentangle energetic contributions from transport and structural effects and clarify when melanin’s protective function shifts toward increased photoreactivity.

## 3. Materials and Methods

### 3.1. Determination of the First Singlet and Triplet Excited States

Melanin, retinal carotenoid and sensitizer models were built in the Avogadro program based on structures found in the literature [21,62]. Models of subunits and dimers were built in different oxidation states: reduced (hydroquinone form—H2Q), partially oxidized (quinone form—IQ) and oxidized (quinone imine forms—QI) (Figure 1). Their ground-state geometry has been optimized at the B3LYP-D4/def2-TZVP in ORCA 6.1 [71], and then reoptimized in other functionals in the same basis set—CAM-B3LYP-D4, r2scan-3c-D4, LC-BLYP-D4, M06-2X and B2PLYP-D4—for comparison. Water solvent was taken into account by means of the implicit CPCM solvation model, used with scaled vdW cavities as implemented in ORCA 6.0.1 software. This model captures the average dielectric response of water, but not explicit hydration or other kinds of local microenvironmental heterogeneity affecting melanin aggregation and excited-state energetics. Subsequent determination of vertical S_1_ and T_1_ energetics was done using Tamm–Dancoff approximation to TD-DFT, and non-equilibrium solvation using infinite ε. For selected systems, adiabatic transitions were calculated using geometries reoptimized in the given functional. Benchmarking of the T1 energetics (presented in ESI, Table S7) revealed that regular B3LYP, together with r2scan-3c, consistently yielded lower transition energies compared to range-separated (LC-BLYP and CAM-B3LYP) and double-hybrid (B2PLYP) functionals, which all produced highly consistent results. By comparison, M06-2X slightly overestimated the triplet energetics. Additionally, we performed DLPNO-STEOM-CCSD/def2-TZVP calculations for DHI-H2Q and DHI-IQ, and arrived at values of vertical triplet energies in agreement with those we are reporting in the text, i.e., 307 kJ/mol and 90 kJ/mol, respectively, compared to CAM-B3LYP values of 339 kJ/mol and 101 kJ/mol, validating the energetic trends, but indicating that caution is necessary when it comes to direct assignment of ^1^O quenching or generation. Unless stated otherwise, CAM-B3LYP energies are used throughout the main text. It is important to note that our protocol yields qualitatively correct triplet energies of photosensitizers such as riboflavin and methylene blue, in good agreement with the experiment (see the relevant Discussion section for details).

One also needs to stress that, despite our attempts to create a balanced and representative set of models of melanin, natural melanins are highly heterogeneous and structurally disordered systems. Consequently, while our choice of representative monomers, dimers, trimers, and tetramers captures key structural and redox motifs, it inherently constitutes a simplified approximation of a complex system.

### 3.2. Characterization of Intermolecular Interactions

Optimized subunits of eu- and pheomelanin were brought together and the possible minima were explored with the Global Optimizer Algorithm (GOAT) implemented in the ORCA 6.01, at the level of XTB [72]. Five of the most optimal (at the XTB level) complexes were reoptimized at the B3LYP-D4/def2-TZVP level for ETS-NOCV analysis, performed in the AMS2024.106 suite, at the B3LYP-D4/TZP level of theory [73].

### 3.3. ETS-NOCV Method

The ETS-NOCV method [74] combines the Extended Transition State (ETS) energy decomposition analysis [75] with the Natural Orbitals for Chemical Valence (NOCV) charge decomposition scheme [76], providing both qualitative and quantitative insight into chemical bonding. Within the ETS approach, the interaction energy between predefined molecular fragments is partitioned into physically interpretable terms:

$$\Delta E_{int}=\Delta E_{orb}+\Delta E_{elstat}+\Delta E_{Pauli}+\Delta E_{disp}$$

Here, ΔE_int_ corresponds to the energy difference between the bonded system and the sum of the energies of its isolated fragments. The first contribution, the orbital term, covers both charge transfer (interactions of occupied orbitals of one fragment with unoccupied orbitals of the other) and polarization effects (intra-fragment charge changes). ΔE_elstat_ represents classical electrostatic attraction between the fragments in the system. The Pauli repulsion contribution, ΔE_Pauli_, describes destabilization arising from interactions between occupied orbitals on different fragments. Finally, ΔE_disp_ is the measure of dispersion forces between the fragments.

The NOCV scheme complements this decomposition by offering an intuitive description of the orbital energy by means of decomposing the deformation density into distinct bonding channels (σ, π, δ, etc.) and associating energetic values with them. It is realized by decomposing the deformation density, $U^{T}\Delta P^{orb}U$ (where ΔP^orb^ is a deformation density matrix expressed on the basis of Lowdin-orthogonalized fragment orbitals (λ), $\chi_{i}=\sum_{j}^{N/2}C_{i,j}\lambda_{j}$). Orbitals created in this way occur in pairs (χ_-i_,χ_i_), which form the said channels (Δρ_k_):

$$\Delta \rho^{orb}=\sum_{k=1}^{N/2}v_{k}\left[-\chi_{-k}^{2}+\chi_{k}^{2}\right]=\sum_{k=1}^{N/2}\Delta \rho_{k}$$

The orbital interaction term ΔE_orb_ can, on the other hand, be written as

$$\Delta E_{orb}=\sum_{\nu }^{N}\sum_{\mu }^{N}\Delta P_{\mu \nu }^{orb}F_{\mu \nu }^{TS}=Tr\left(\Delta \rho^{orb}F^{TS}\right)$$

where FTS is a Fock matrix of the system in the “transition state”, in which the electron density is a mix of that of the molecule and promolecules: *ρ^TS^* = 1/2*ρ* + 1/2*ρ*^0^. The equation above can be rewritten in terms of energies corresponding to the density channels delta-rho-orb:

$$\Delta E_{orb}=\sum_{k=1}^{N/2}v_{k}\left[-F_{-k,-k}^{TS}+F_{k,k}^{TS}\right]=\sum_{k=1}^{N/2}\Delta E_{k}^{orb}$$

## 4. Conclusions

The results of our study show that the triplet-state energetics of eumelanin are primarily determined by its oxidation state, with a clear difference between the low T_1_ energies of oxidized forms (QI, IQ) and the much higher T_1_ values of their reduced (H2Q) counterparts. Notably, the T_1_ energies of oxidized eumelanin are found to be comparable to, and in some cases lower than, those of carotenoids, typically viewed as natural pigments with the lowest triplet manifolds, indicating that oxidized eumelanin is energetically well-suited to accept low-energy triplet excitations. The energy range of the first triplet excited state in oxidized eumelanins is particularly consequential in the retina, where eumelanin is exposed to chronic photo-oxidative stress, highly polyunsaturated lipids and photosensitizing agents such as lipofuscin and modified retinoids [77,78]. Under these conditions, the low T_1_ levels of eumelanin suggest thermodynamic feasibility for accepting triplet energy from such chromophores.

Although detailed pathways of electronic and physical deactivation require further investigation, these important energetics establish a functional parallel to the triplet-quenching behavior of carotenoids, suggesting a protective role of RPE melanin based on its excited-state quenching capability. In contrast, structural units of pheomelanin are dominated by high T_1_ energies. Only oxidized benzothiazole derivatives and trichochromes exhibit triplet levels energetically compatible with singlet oxygen quenching. However, these values remain substantially higher than for oxidized eumelanins, implying intrinsically lower quenching potential. Supramolecular analysis highlights a clear contrast in aggregation, which might lead to different solvation dynamics in the condensed phase: eumelanin assemblies are stabilized by a balance of π–π stacking and hydrogen bonding, whereas pheomelanin assemblies are dominated by dense hydrogen-bond networks. Furthermore, ETS-NOCV analyses revealed that the cooperative action of electrostatics, charge delocalization and London dispersion forces in pheomelanin aggregates leads to cumulative stabilization energies exceeding 100 kcal/mol, i.e., values comparable in magnitude to strong covalent bond energies [79].

## Figures and Tables

**Table 1:** Comparison of T_1_ → S_0_ transition values for selected eumelanin and pheomelanin models, selected retinal carotenoids (lutein, zeaxanthin) and selected sensitizers (methylene blue, riboflavin, sorbic alcohol). ch in the name denotes charged species (−1 charge).

System			Vertical Transition [kJ/mol]	Adiabatic Transition [kJ/mol]
**Monomers**				
**Eumelanin**	H2Q	DHI	339	294
		DHICA	289	244
		DHICA ch	311	x
	IQ	DHI	101	62
		DHICA	96	55
		DHICA ch	89	x
	QI	DHI	117	28
		DHICA	118	33
		DHICA ch	112	x
**Pheomelanin**	Benzothiazine subunits	BT	306	217
		BTCA	265	175
		ODHBT	353	291
		DHBTCA	362	296
	Benzothiazole subunits	BZ	345	290
		BZox	132	84
**Dimers**				
**Eumelanin**	H2Q	22 DHI	272	236
		24 DHI	306	243
		27 DHI	288	242
		27 DHI (NH)	306	252
		44 DHICA	282	241
	IQ	22 DHI	90	55
		24 DHI	78	22
		27 DHI	96	45
		44 DHICA	88	43
	QI	22 DHI	92	66
		24 DHI	109	15
		27 DHI	110	32
		44 DHICA	82	36
	H2Q-IQ	22 DHI	100	x
		24 DHI	71	x
		27 DHI	84	x
**Pheomelanin**	BTCA product oxidation	Trichochrome 1	206	80
		Trichochrome 2	168	75
		BT-TIQ	306	267
	BT product oxidation	Dimer	346	291
		Cyclo	349	293
**Trimers**				
**Eumelanin**	H2Q	Trimer DHI	267	231
		Trimer DHICA	280	249
	IQ	Trimer DHI	90	6
		Trimer DHICA	93	47
	QI	Trimer DHI	106	24
		Trimer DHICA	119	38
**Other structures**				
**Retinal carotenoids**		Lutein	x	57
		Zeaxantin	x	64
**Sensitizers**		Methylene blue	138	120
		Riboflavin	227	195
		Sorbic alcohol	317	226

## Data Availability

Data are available from the authors on reasonable request.

## Supplementary Materials

The following supporting information can be downloaded at https://www.mdpi.com/article/10.3390/ijms27135886/s1.

## Abbreviations

The following abbreviations are used in this manuscript:

AHP	Aminohydroxyphenylalanine
ALA	Alanine
ASP	Agouti Signaling Protein
BT	2-amino-2-carboxyethyl)-4-hydrobenzothiazine
BT-TIQ	Dimeric Benzothiazolylthiazinodihydroisoquinoline Intermediate
BTCA	7-(2-Amino-2-carboxyethyl)-5-hydroxy-2H-1,4-benzothiazine-3-carboxylic Acid
BZ	6-(2-Amino-2-carboxyethyl)-4-hydroxybenzothiazole
BZox	6-(2-Amino-2-carboxyethyl)-4-oxo-4H-1,3-benzothiazole
DHI	5,6-Dihydroxyindole
DHBTCA	7-(2-Amino-2-carboxyethyl)-1,4-dihydrobenzothiazine-3-carboxylic Acid
DHICA	5,6-Dihydroxyindole-2-carboxylic Acid
EPR	Electron Paramagnetic Resonance
ETS	Extended Transition State
GOAT	Global Optimizer Algorithm
H2Q	Hydroquinone
IQ	Quinone
ISC	Intersystem Crossing
L-DOPA	3,4-Dihydroxyphenylalanine
NOCV	Natural Orbitals for Chemical Valence
ODHBT	7-(2-Amino-2-carboxyethyl)-5-hydroxy-3-oxo-3,4-dihydro-2H-1,4-benzothiazine
PDCA	Pyrrole-2,3-dicarboxylic Acid
PMEL17	Premelanosome Protein 17
PTCA	Pyrrole-2,3,5-tricarboxylic Acid
PTeCA	Pyrrole-2,3,4,5-tetracarboxylic Acid
QI	Quinone Imine
RPE	Retinal Pigment Epithelium
ROS	Reactive Oxygen Species
S_0_	Ground State (Singlet)
S_1_	First Excited Singlet State
T_1_	First Excited Triplet State
TD-DFT	Time-Dependent Density Functional Theory
TDCA	Thiazole-4,5-dicarboxylic Acid
TTCA	Thiazole-2,4,5-tricarboxylic Acid
UV	Ultraviolet
