# Supplementary material for: From Monomers to Aggregates: The Influence of Redox State and Structure on the First Excited States of Eumelanin and Pheomelanin
Source: Int J Mol Sci. 2026 Jun 30;27(13):5886. doi: 10.3390/ijms27135886 (PMC13361419; doi:10.3390/ijms27135886)
Supplement: Supplementary file 1 [file ijms-27-05886-s001.zip › ijms-4335804-supplementary.pdf]

Supporting Information:

From Monomers to Aggregates: The Influence of Redox State and Structure on the First Excited States of Eumelanin and Pheomelanin

Joanna Waresiak[1,3], **Filip Sagan**[2], Mariusz Mitoraj[2], Tadeusz Sarna[1]

[1] Department of Biophysics, Faculty of Biochemistry, Biophysics and Biotechnology, Jagiellonian University, Gronostajowa 7, 30-387 Krakow, Poland

[2] Department of Computational Methods in Chemistry, Faculty of Chemistry, Jagiellonian University, Gronostajowa 2, 30-387 Krakow, Poland

[3] Doctoral School of Exact and Natural Sciences, Jagiellonian University, Lojasiewicza 11, 30-348 Krakow, Poland

Comparison of ground and excited state geometries

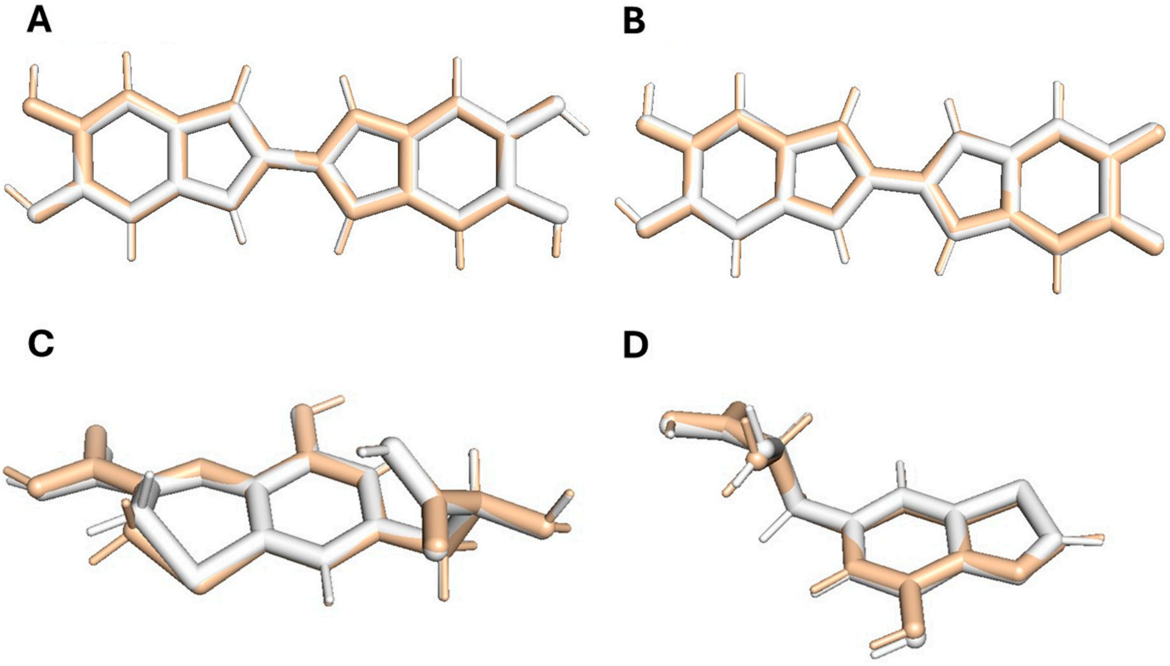

Figure S1. Superimposition of the ground state (white) and first excited singlet state (wheat) geometries of the eumelanin 22-DHI dimer in the reduced state (A) and the mixed dimer (B) and the benzothiazine (C) and benzothiazole (D) subunits

Geometry considerations

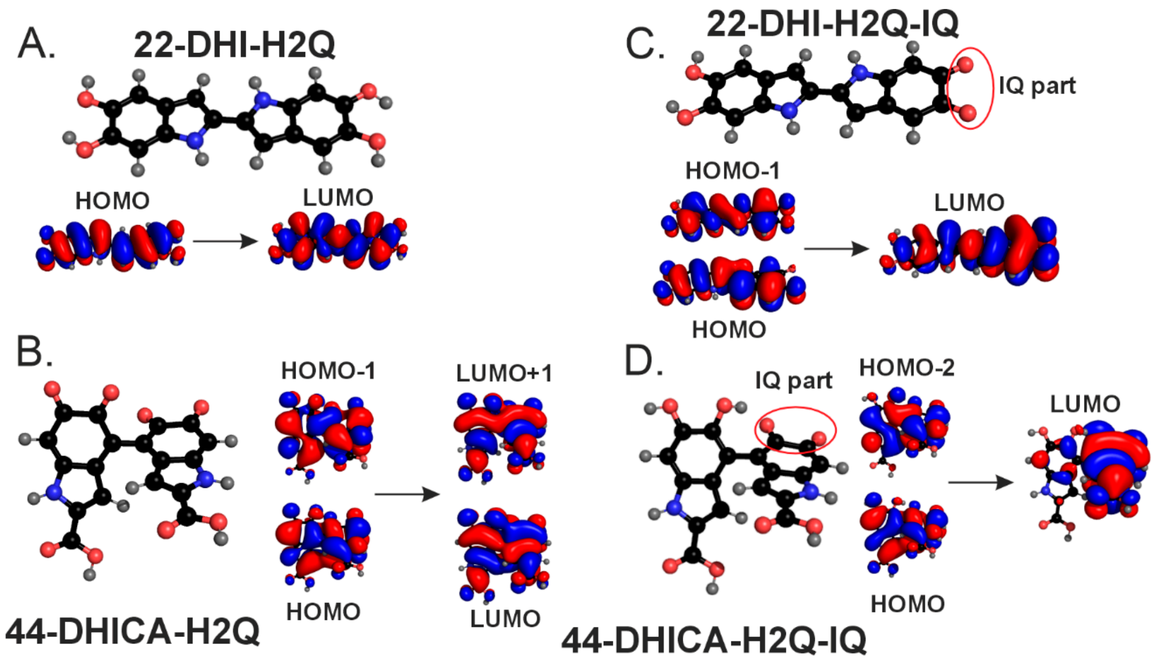

Figure S2. The most important MO contributions to the S0->T1 transitions featuring well-delocalized triplet excitations for A. 22-DHI-H2Q dimer and B. 44-DHICA-H2Q dimer compared to significantly more localized counterparts for systems of mixed oxidation state: C. 22-DHI-H2Q-IQ and D. 44-DHICA-H2Q-IQ.

Table S1. Mean of interaction energies for systems containing DHI or DHICA molecules in different oxidation states. Values are computed across all pairwise interactions, calculated at the B3LYP-D4/TZP level of theory.

	Mean (kcal/mol)		Std. Error	
	**DHI**	**DHICA**	**DHI**	**DHICA**
**H2Q**	−17.68	−20.25	4.43	4.62
**IQ**	−17.59	−19.46	2.96	2.90
**QI**	−17.16	−19.27	3.00	3.09

Table S2. Means of interaction energies for systems containing different pheomelanin subunits. Values are computed across all pairwise interactions, calculated at the B3LYP-D4/TZP level of theory.

	**Mean (kcal/mol)**	**Std. Error**
**BT**	−39.34	1.50
**BZ**	−40.75	1.80
**2_BTCA**	−49.72	1.69
**ODHBT**	−53.76	1.60
**DHBTCA**	−48.84	2.65

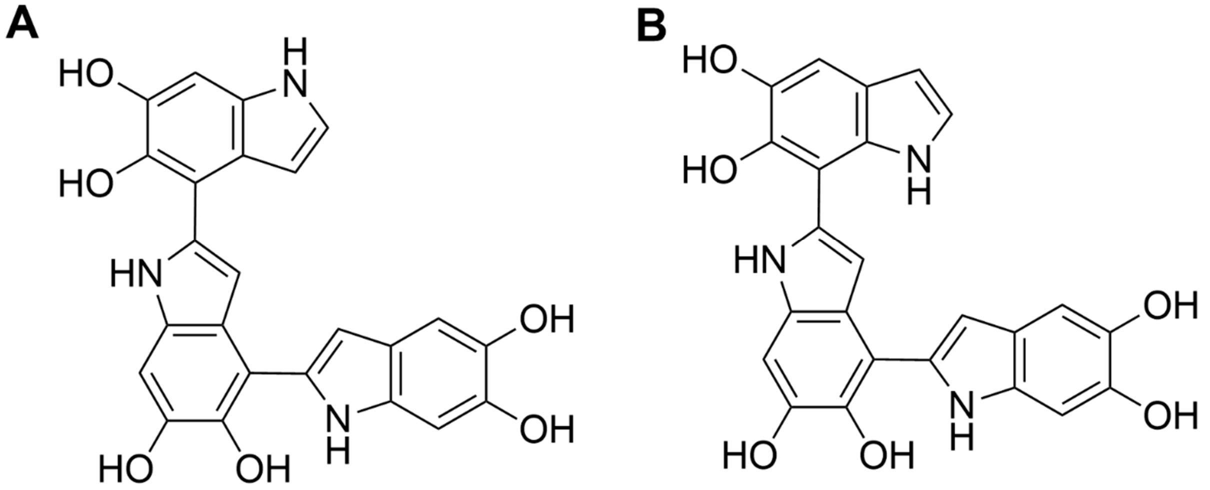

Figure S3. Covalently bonded DHI trimers. Presented are H2Q (hydroxyquinone) species: (A) 42-42 trimer and (B) 72-42 trimer.

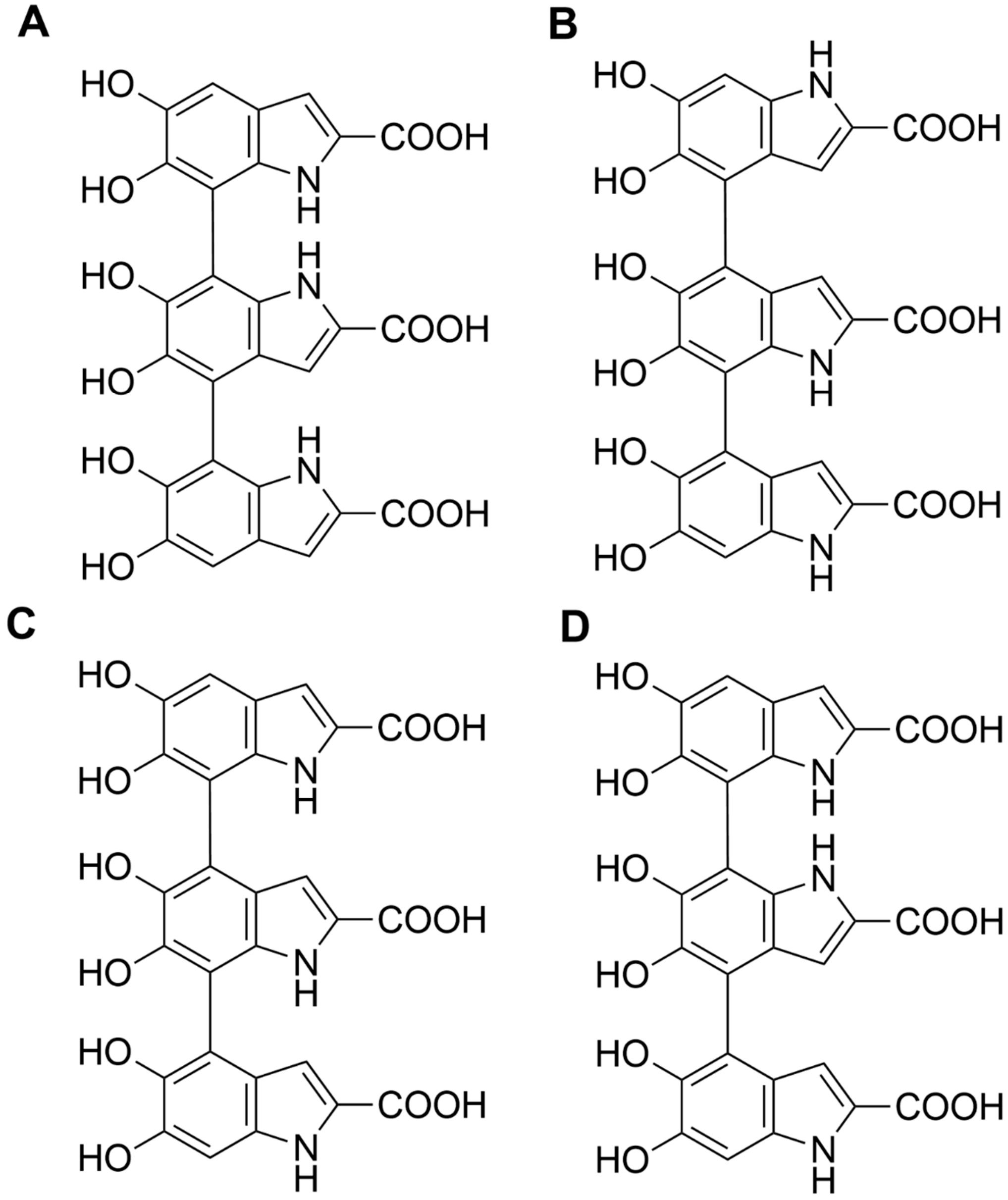

Figure S4. Covalently bonded DHICA trimers. Presented are H2Q (hydroxyquinone) species: (A) 74-47 trimer (B) 44-74 trimer, (C) 74-74 trimer (D) 77-44 trimer.

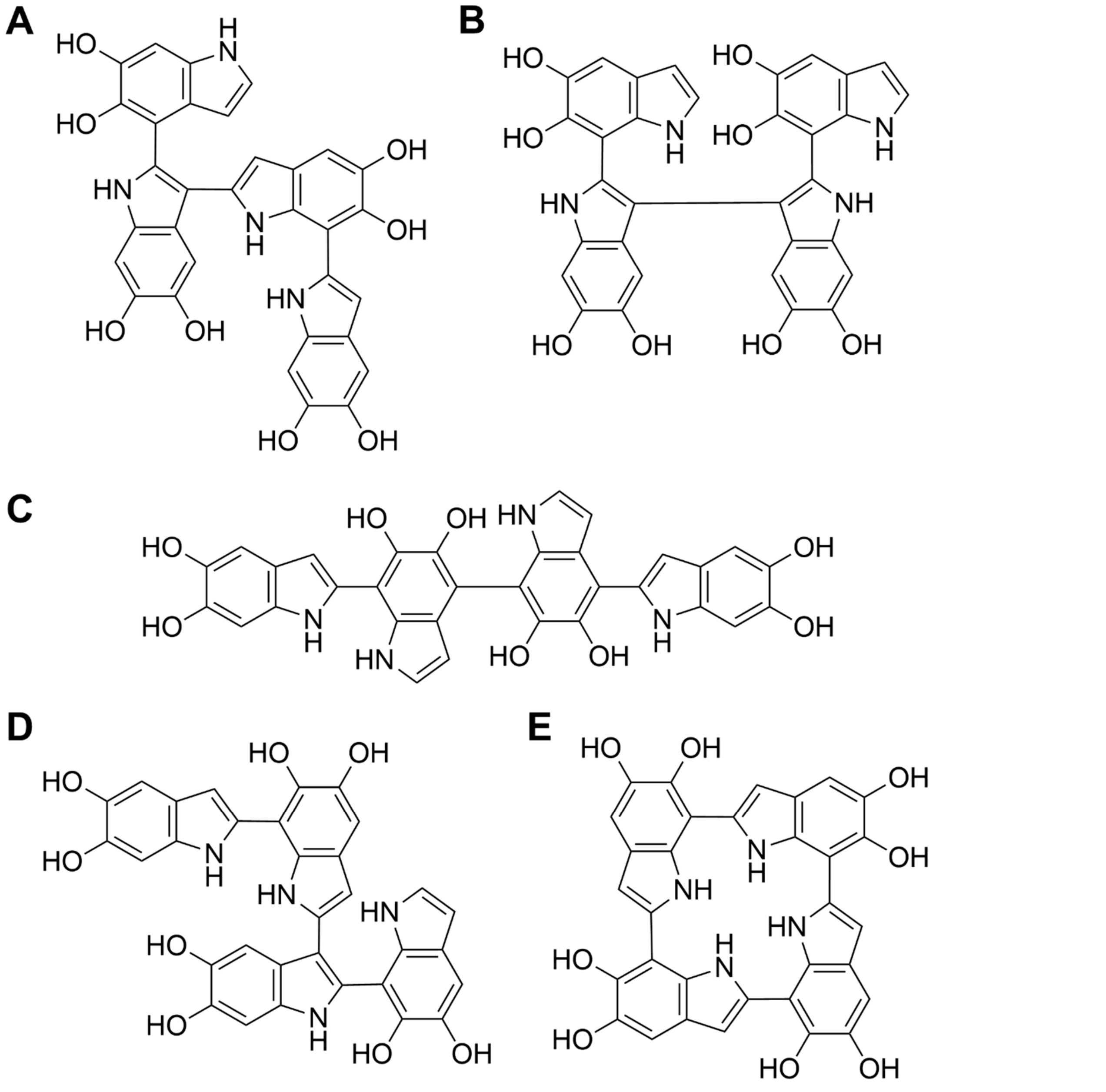

Figure S5. Covalently bonded DHI tetramers. Presented are H2Q (hydroxyquinone) species: (A) 24-23-24 tetramer, (B) 72-33-27 tetramer, (C) 27-44-72 tetramer and (D) 27-23-27 tetramer.

DHI and DHICA-based trimers and tetramers

To address the structural complexity of melanin, we expanded our investigation to assess how increasing oligomer size influences the tested properties. Due to the sheer number of possibilities in constructing the polymeric strictures of eumelanin, we limited our studies to just a few selected covalently-bonded structures. In the case of DHI, the 42-42 homo-trimer (i.e. three DHI monomers, connected by 4 and 2 positions in both connection sites) in all three oxidation states, and the 72-42 trimer in all three oxidation states were chosen, (Figure S3 in SI). In the case of DHICA, the systems selected for studies were: 74-

47 trimer, 44-74 trimer, 74-74 trimer, and 77-44 trimer, (Figure S4 in SI). Conversely, four DHI tetramers were built and studied: 24-23-24 tetramer, 72-33-27 tetramer, 27-44-72 tetramer and 27-23-27 tetramer. Additionally, a postulated closed structure of heme-like 27-27-27-27-cyclotetramer was built. All structures are also presented in the SI, (Figure S5 in SI). Excited-state analysis of eumelanin trimers, (Figure S6) confirmed the persistence of two distinct energy fractions, corresponding to the reduced and oxidized forms, consistent with the trends observed in monomers, (main text, Figure 2) and dimers, (main text, Figure 4). Despite the increased molecular size, oxidation state remained the dominant factor influencing both the absorption properties and the energies of the triplet states. An analogous tendency was observed for the DHI tetramers, whose excited-state energies and overall photophysical behavior remained closely aligned with those of the DHI trimers (Table S4 in SI).

DHICA trimers exhibited minimal excited-state energy changes compared to their dimeric counterparts. This reflects the inherently more rigid structure of DHICA, facilitating further delocalization upon oligomer elongation.

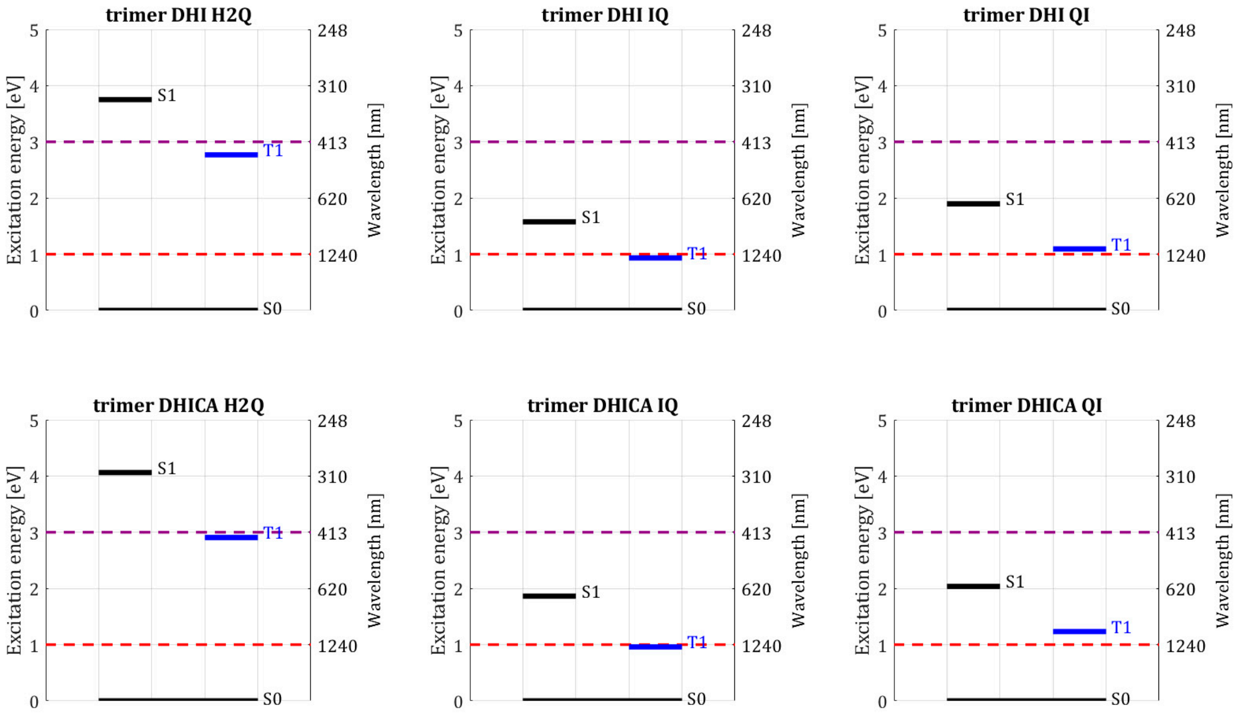

Figure S6. Location of the first triplet (T_1) and singlet (S_1) states for eumelanin trimer DHI and DHICA in other redox state (H2Q, IQ, QI). The values determined as a vertical transition are presented **(S_1 in black, T_1 in blue).** Additionally, the red line is the boundary of singlet oxygen phosphorescence, and the purple line is the boundary associated with the light that is able to reach the retina (below this line)

Energy diagrams

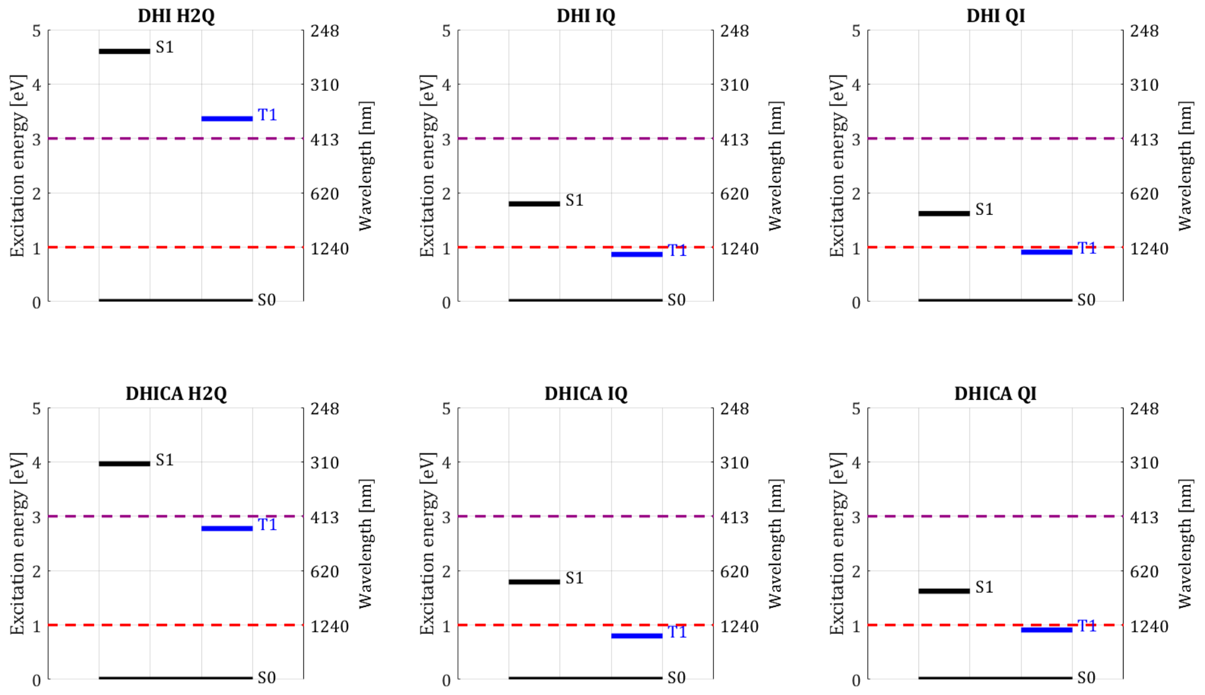

Figure S7. Location of the first triplet (T_1) and singlet (S_1) states for eumelanin subunit DHI and DHICA in other redox state (H2Q, IQ, QI). The values determined as a vertical transition using B3LYP are presented. Additionally, the red line is the boundary of singlet oxygen phosphorescence, and the purple line is the boundary associated with the light that is able to reach the retina (below this line)

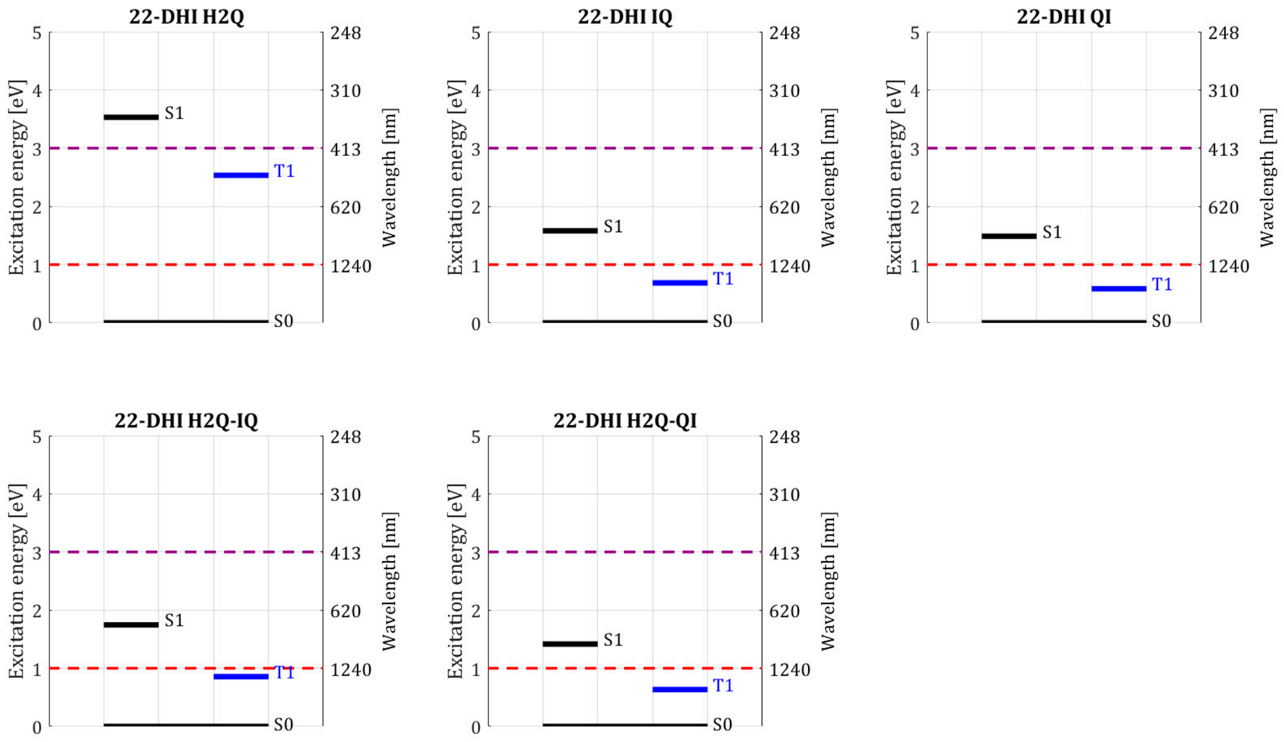

Figure S8. Location of the first triplet (T_1) and singlet (S_1) states for eumelanin dimer 22-DHI in other redox state (H2Q, IQ, QI) and mix (H2Q-IQ, H2Q-QI). The values determined as a vertical transition using B3LYP are presented. Additionally, the red line is the boundary of singlet oxygen phosphorescence, and the purple line is the boundary associated with the light that is able to reach the retina (below this line)

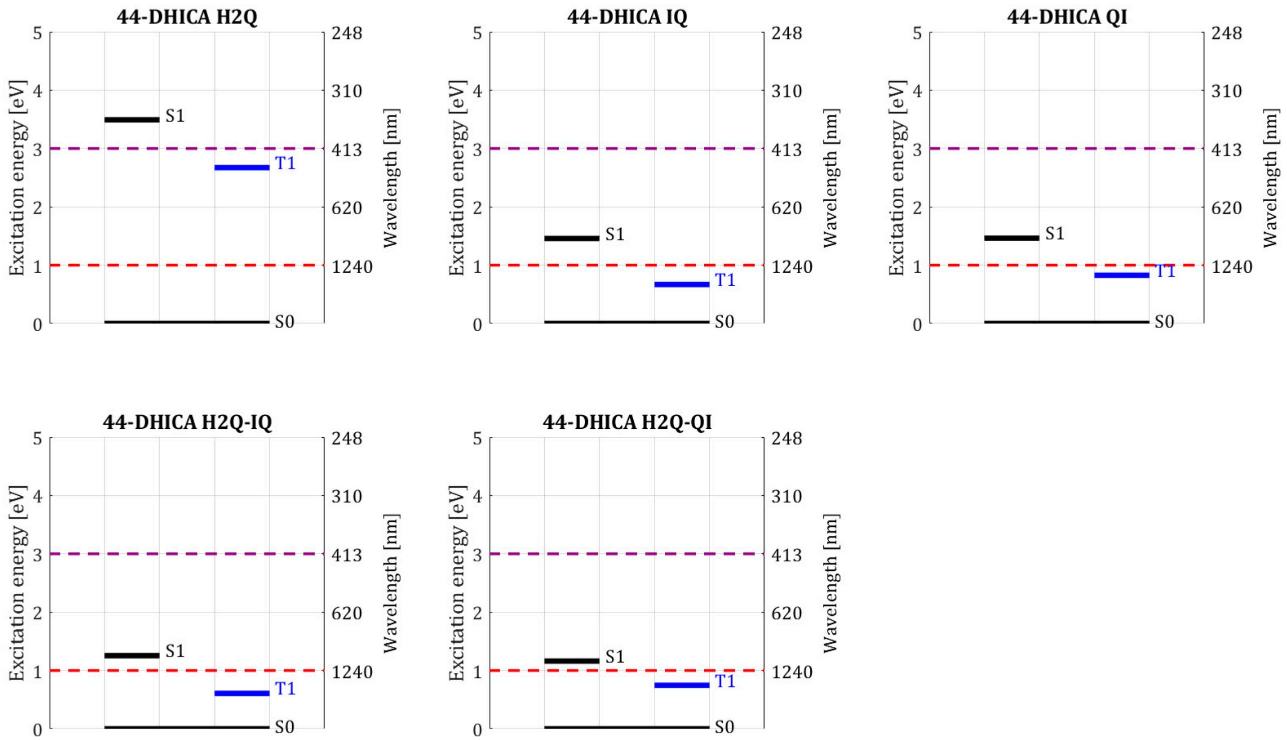

Figure S9. Location of the first triplet (T_1) and singlet (S_1) states for eumelanin dimer 44-DHICA in other redox state (H2Q, IQ, QI) and mix (H2Q-IQ, H2Q-QI). The values determined as a vertical transition using B3LYP are presented. Additionally, the red line is the boundary of singlet oxygen phosphorescence, and the purple line is the boundary associated with the light that is able to reach the retina (below this line)

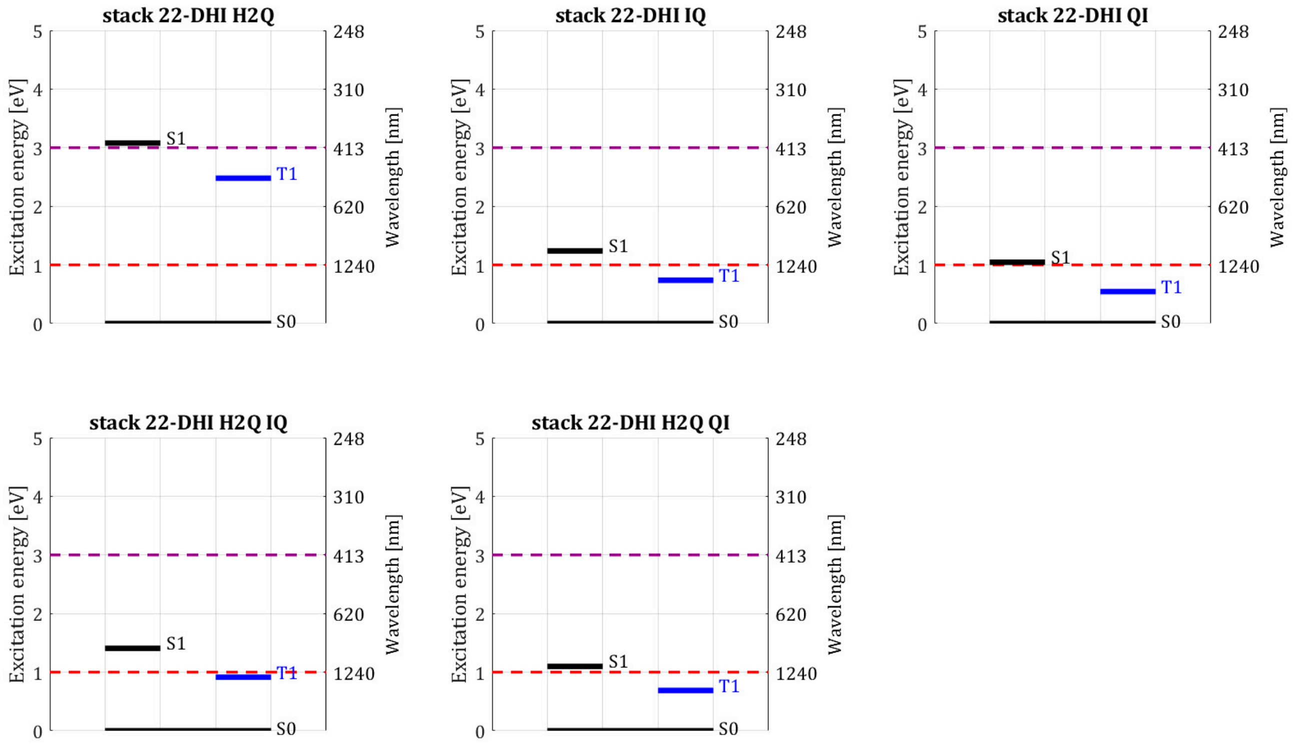

Figure S10. Location of the first triplet (T_1) and singlet (S_1) states for 22 DHI stack in different redox state. The values determined as a vertical transition using B3LYP are presented. Additionally, the red line is the boundary of singlet oxygen phosphorescence, and the purple line is the boundary associated with the light that is able to reach the retina (below this line)

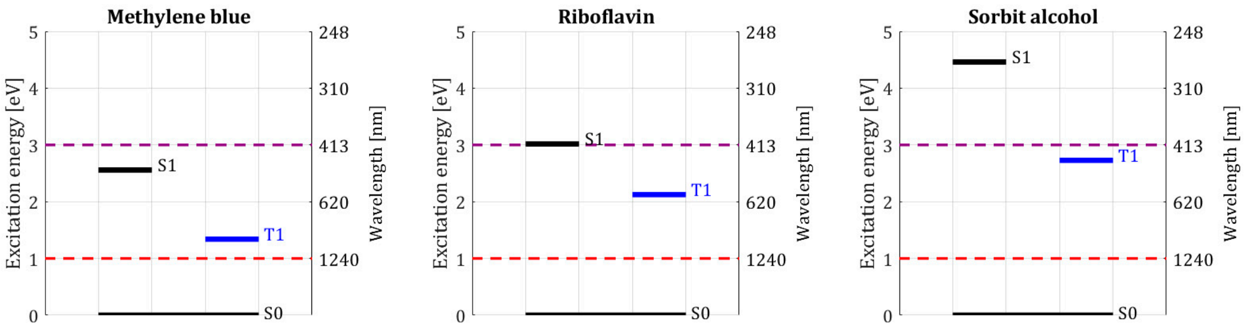

Figure S11. Location of the first triplet (T_1) and singlet (S_1) states for methylene blue, riboflavin and sorbic alcohol. The values determined as a vertical transition using B3LYP are presented. Additionally, the red line is the boundary of singlet oxygen phosphorescence, and the purple line is the boundary associated with the light that is able to reach the retina (below this line)

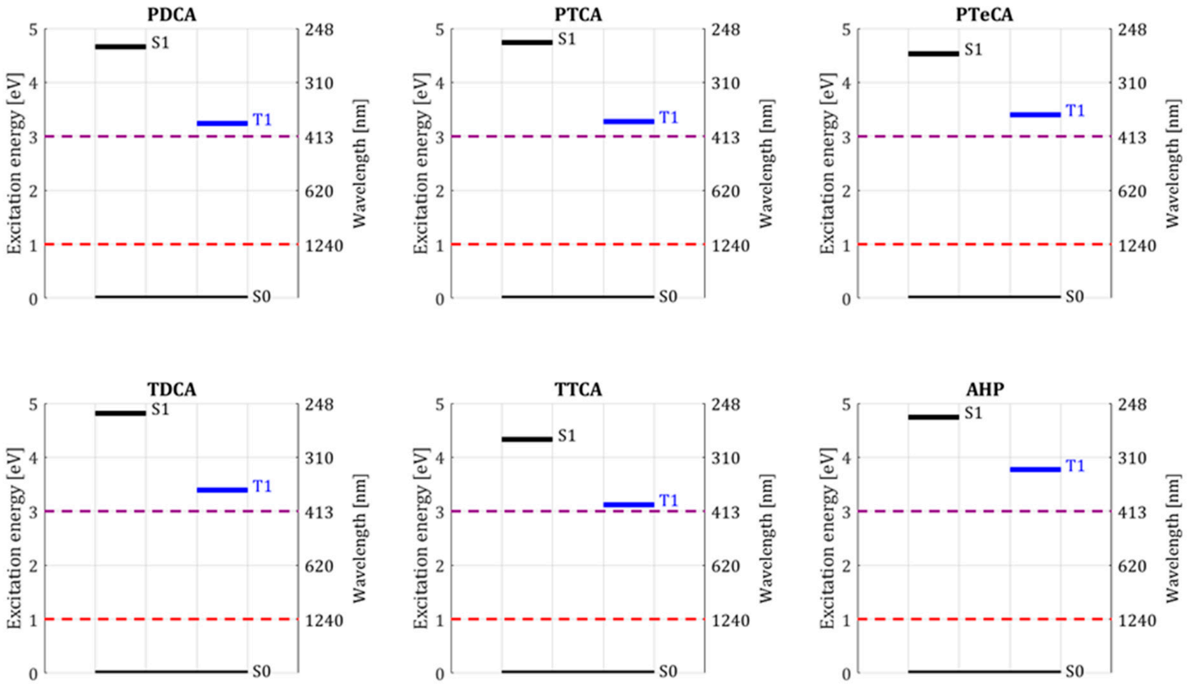

Figure S12. Location of the first triplet (T_1) and singlet (S_1) states for PDCA, PTCA, PTeCA, TDCA, TTCA, AHP. The values determined as a vertical transition using B3LYP are presented. Additionally, the red line is the boundary of singlet oxygen phosphorescence, and the purple line is the boundary associated with the light that is able to reach the retina (below this line)

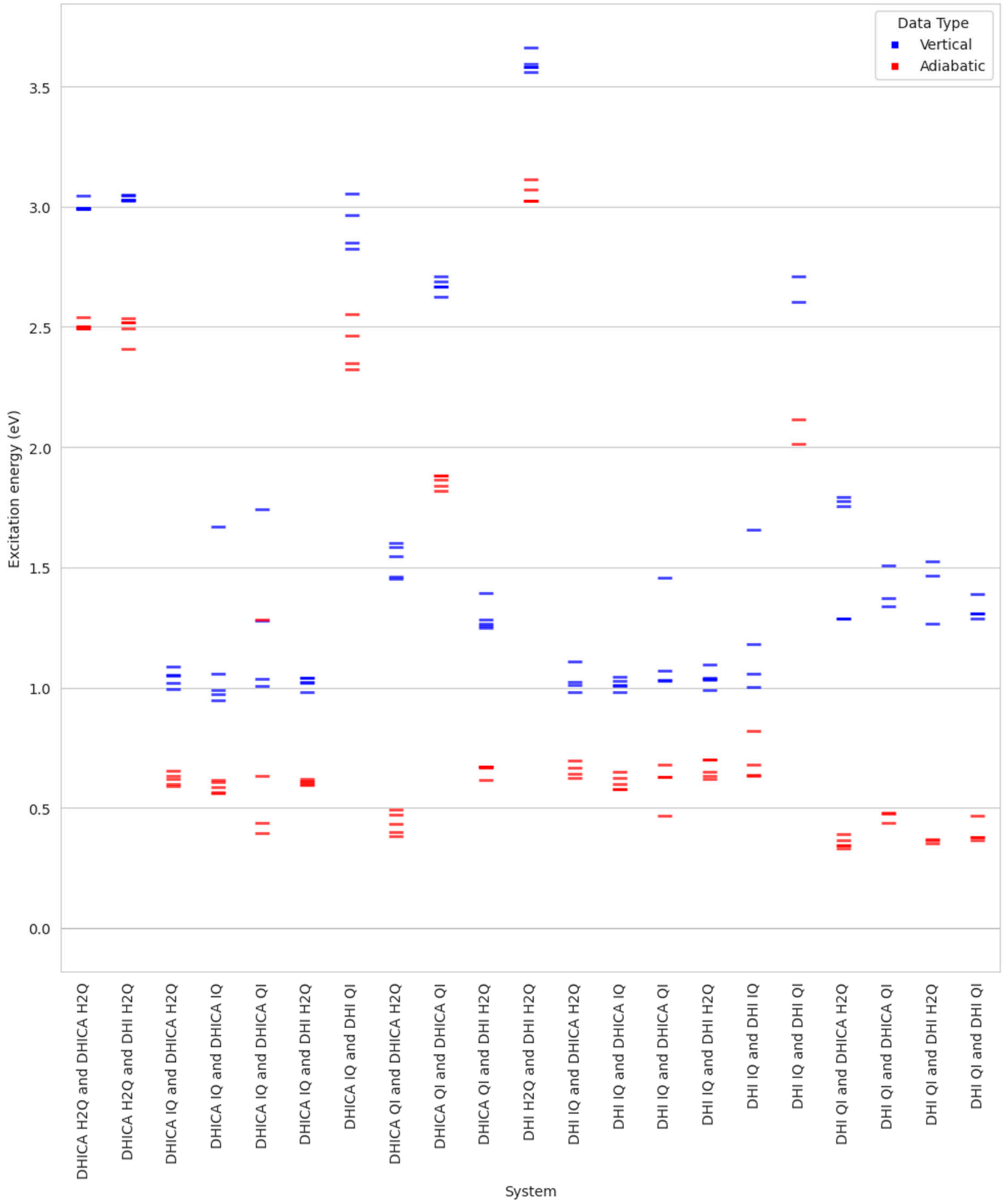

Figure S13. Location of the first triplet (T_1) states in the vertical (blue) and adiabatic (red) approaches, for non-covalently bonded dimers of eumelanin composed of DHI and DHICA subunits. Each bar corresponds to one of the obtained minima for a given system.

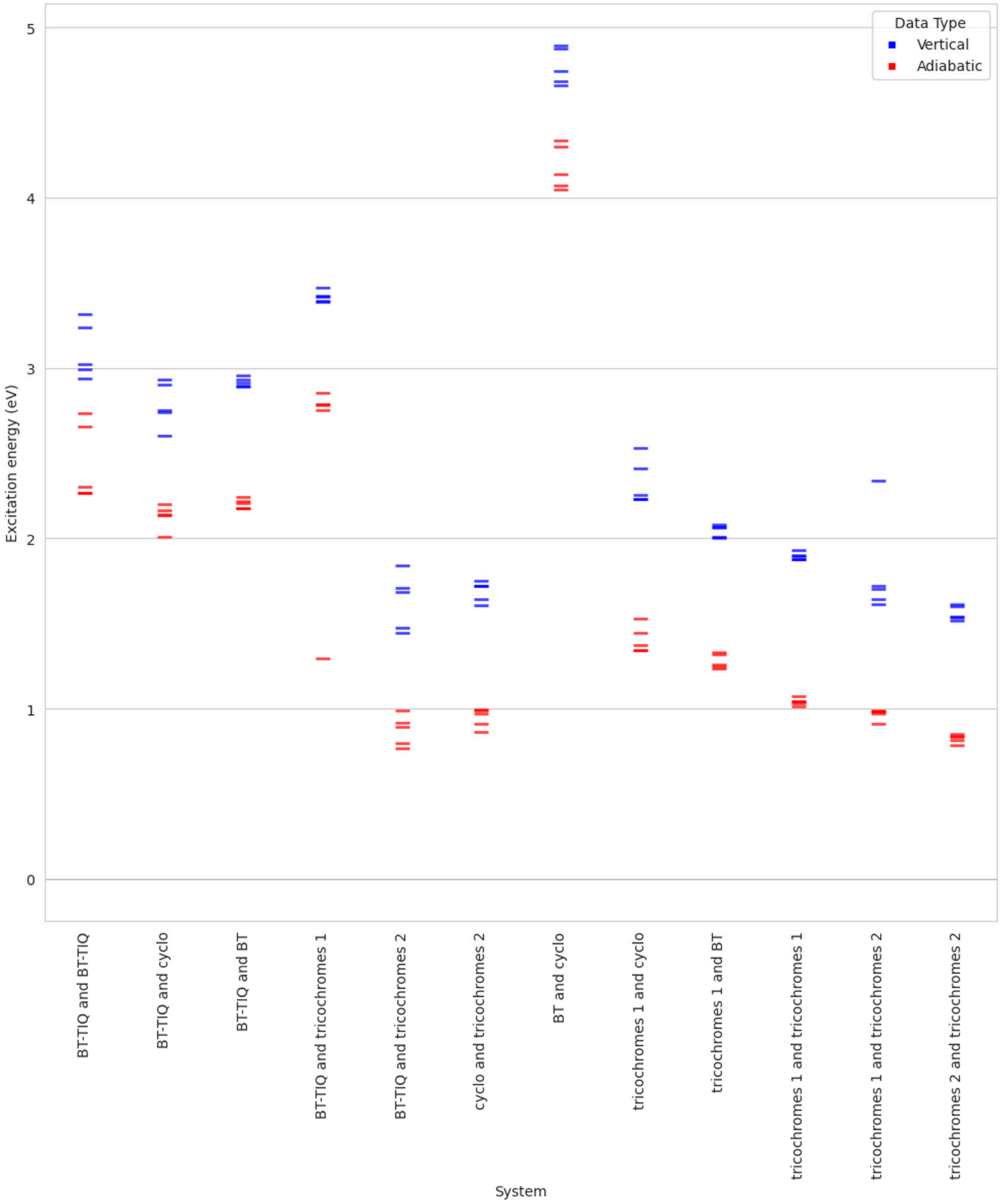

Figure S14. Location of the first triplet (T_1) states in the vertical (blue) and adiabatic (red) approaches, for complexes of dimeric pheomelanin systems: BT-TIQ, both discussed tricochromes, cyclo system, and regular BT dimer. Each bar corresponds to one of the obtained minima for a given system.

Determination of the first singlet and triplet excited states

Vertical transition

Table S3. Summary of calculated energies (kJ/mol) of electronic transitions for selected eumelanin (DHI, DHICA) and pheomelanin (BT, BTCA, ODHBT, DHBTCA, 5BZ_ox) subunits. The $S_0 \rightarrow S_1$ transition corresponds to absorption, fluorescence, or internal conversion; $S_1 \rightarrow T_1$ represents intersystem crossing; and $T_1 \rightarrow S_0$ corresponds to phosphorescence or internal conversion. All calculations were performed using time-dependent density functional theory (TD-DFT) at the B3LYP/CAM-B3LYP/r2scan-3c/def2-TZVP level in water

		**Type of transition (kJ/mol)**								
		**$S_1 \rightarrow S_0$**			**$S_1 \rightarrow T_1$**			**$T_1 \rightarrow S_0$**		
**System**		B3LYP	CAM-B3LYP	r2scan-3c	B3LYP	CAM-B3LYP	r2scan-3c	B3LYP	CAM-B3LYP	r2scan-3c
**Eumelanin**	DHI H2Q	444	469	440	120	130	125	324	339	315
	DHI IQ	173	200	166	90	99	92	84	101	74
	DHI QI	156	198	136	68	81	71	88	117	65
	DHICA H2Q	382	429	360	115	139	103	268	289	256
	DHICA IQ	173	201	158	96	105	92	77	96	66
	DHICA QI	157	200	134	69	83	71	88	118	63
**Pheomelanin**	5 BTCA	287	351	260	52	86	41	235	265	220
	5 BT	347	397	328	65	92	61	282	306	267
	5 ODHBT	412	456	397	79	103	77	333	353	321
	5 DHBTCA	427	476	391	80	113	59	347	362	332
	5 BZ	420	464	395	93	119	83	326	345	312
	5 BZox	117	215	92	9	84	68	108	132	24
	2 BTCA	286	352	254	48	84	41	237	268	213
	2 BT	357	406	336	63	89	58	294	317	278
	2 ODHBT	417	460	399	78	103	73	338	357	326
	2 DHBTCA	440	457	386	90	115	62	350	364	337
	2 BZ	413	478	399	90	115	79	322	343	308

Table S4. Summary of calculated energies (kJ/mol) of electronic transitions for selected eumelanin dimers (DHI, DHICA) and pheomelanin. The $S_0 \rightarrow S_1$ transition corresponds to absorption, fluorescence, or internal conversion; $S_1 \rightarrow T_1$ represents an intersystem transition; and $T_1 \rightarrow S_0$ corresponds to phosphorescence or internal conversion. All calculations were performed using time-dependent density functional theory (TD-DFT) at the B3LYP/CAM-B3LYP/r2scan-3c/def2-TZVP level in water

		**Type of transition (kJ/mol)**								
		**$S_1 \rightarrow S_0$**			**$S_1 \rightarrow T_1$**			**$T_1 \rightarrow S_0$**		
**System**		B3LYP	CAM-B3LYP	r2scan-3c	B3LYP	CAM-B3LYP	r2scan-3c	B3LYP	CAM-B3LYP	r2scan-3c
**Eumelanin**	22 DHI H2Q	341	381	324	96	109	96	244	272	228
	2,2 DHI IQ	153	197	111	86	107	63	66	90	48
	2,2 DHI QI	143	183	114	87	91	88	56	92	27
	2,2 DHI mix H2Q + IQ	168	193	161	86	93	84	83	100	77
	2,2 DHI mix H2Q + QI	137	171	125	75	76	73	61	95	53
	2,4 DHI H2Q	348	393	330	93	87	88	255	306	242
	2,4 DHI IQ	112	167	74	60	89	47	52	78	27
	2,4 DHI QI	134	189	107	64	80	72	70	109	35
	2,4 DHI mix H2Q + IQ	125	159	121	84	87	94	41	71	28
	2,4 DHI mix H2Q + QI	132	172	114	72	77	71	59	95	43
	2,7 DHI H2Q	358	404	339	96	116	90	262	288	249
	2,7 DHI IQ	135	187	90	66	92	43	69	96	47
	2,7 DHI QI	135	192	92	64	82	51	71	110	41
	2,7 DHI mix H2Q + IQ	128	170	126	76	86	88	52	84	37
	2,7 DHI mix H2Q + QI	119	182	100	55	71	67	64	111	33
	4,4 DHICA H2Q	337	404	137	79	122	47	258	282	251
	4,4 DHICA IQ	141	X	298	76	x	58	65	X	46
	4,4 DHICA QI	141	X	104	61	X	46	80	X	53
	4,4 DHICA mix H2Q + IQ	121	X	99	62	X	31	59	X	40
	4,4 DHICA mix H2Q + QI	112	X	71	40	X	24	72	X	46
	4,7 DHICA H2Q	341	X	295	83	X	51	258	X	244
	4,7 DHICA IQ	143	X	101	75	X	52	68	X	50
	4,7 DHICA QI	143	X	82	61	X	26	82	X	56
	4,7 DHICA mix H2Q + IQ	130	X	83	58	X	28	72	X	55
	4,7 DHICA mix H2Q + QI	120	X	78	37	X	21	83	X	57
	7,7 DHICA H2Q	351	X	299	86	X	48	265	X	251
	7,7 DHICA IQ	146	X	103	74	X	49	72	X	54
	7,7 DHICA QI	151	X	100	63	X	37	88	X	63
	7,7 DHICA mix H2Q + IQ	131	x	77	58	X	22	73	X	55
	7,7 DHICA mix H2Q + QI	111	X	53	0	X	8	111	X	45
**Pheomelanin**	cyclo	405	452	346	74	103	30	331	349	316
	dimer	414	445	353	81	98	37	333	346	316

	BT-TIQ	310	392	263	43	86	31	267	306	231
	tricochromes_1	255	307	236	77	101	73	178	206	164
	tricochromes_2	226	273	214	93	104	95	133	168	120

Table S5. Summary of calculated energies (kJ/mol) of electronic transitions for selected eumelanin trimers and tetramers. The transition $S_0 \rightarrow S_1$ corresponds to absorption, fluorescence, or internal conversion; $S_1 \rightarrow T_1$ represents an intersystem transition; and $T_1 \rightarrow S_0$ corresponds to phosphorescence or internal conversion. All calculations were performed using time-dependent density functional theory (TD-DFT) at the B3LYP/CAM-B3LYP/r2scan-3c/def2-TZVP level in water

		**Type of transition (kJ/mol)**								
		**$S_1 \rightarrow S_0$**			**$S_1 \rightarrow T_1$**			**$T_1 \rightarrow S_0$**		
**System**		B3LYP	CAM-B3LYP	r2scan-3c	B3LYP	CAM-B3LYP	r2scan-3c	B3LYP	CAM-B3LYP	r2scan-3c
**Trimers**	DHI_trimer_1_H2Q	305	446	276	69	179	56	236	267	220
	DHI_trimer_1_IQ	72	152	52	23	62	47	49	90	4
	DHI_trimer_1_QI	119	183	85	60	77	72	59	106	13
	DHICA_trimer_1_H2Q	320	392	279	72	112	58	246	280	230
	DHICA_trimer_1_IQ	131	180	88	36	87	36	54	93	14
	DHICA_trimer_1_QI	143	197	80	64	78	61	52	119	12
**Tetramers**	DHI_tetramer_7_H2Q	305	X	264	60	X	42	245	X	223
	DHI_tetramer_7_IQ	84	X	41	22	X	26	62	X	15
	DHI_tetramer_7_QI	100	X	67	72	X	58	28	X	9
	DHI_tetramer_8_H2Q	328	X	286	66	X	47	262	X	238
	DHI_tetramer_8_IQ	90	X	56	28	X	19	62	X	37
	DHI_tetramer_8_QI	95	X	37	40	X	22	55	X	16
	DHI_tetramer_9_H2Q	319	X	269	61	X	37	258	X	232
	DHI_tetramer_9_IQ	110	X	68	54	X	39	56	X	29
	DHI_tetramer_9_QI	125	X	63	56	X	29	69	X	34
	DHI_tetramer_10_H2Q	323	X	286	70	X	54	253	X	231
	DHI_tetramer_10_IQ	109	X	44	41	X	2	69	X	42
	DHI_tetramer_10_QI	93	X	44	56	X	43	37	X	1
	DHI_tetramer_hem_H2Q	321	X	286	76	X	54	244	X	231
	DHI_tetramer_hem_IQ	123	X	44	74	X	2	49	X	42
	DHI_tetramer_hem_QI	102	X	44	44	X	43	57	X	X

Adiabatic transition

Table S6. Summary of calculated energies (kJ/mol) of electronic transitions for selected eumelanin and pheomelanin structures. Where the $T_1 \rightarrow S_0$ transition corresponds to phosphorescence or internal conversion. All calculations were performed as adiabatic transitions at the B3LYP/CAM-B3LYP/r2scan-3c/def2-TZVP level in water

			**$T_1 \rightarrow S_0$ (kJ/mol)**	
		**Monomers**		
**System**		B3LYP	CAM-B3LYP	r2scan-3c
**Eumelanin**	DHI H2Q	288	294	275
	DHI IQ	57	62	47
	DHI QI	24	28	10
	DHICA H2Q	236	244	226
	DHICA IQ	50	55	38
	DHICA QI	27	33	12
**Pheomelanin**	5 BTCA	174	175	159
	5 BT	211	217	193
	5 ODHBT	240	291	234
	5 DHBTCA	287	296	289
	5 BZ	283	290	268
	2 BTCA	181	177	160
	2 BT	217	222	198
	2 ODHBT	241	300	239
	2 BZ	280	288	285
		**Dimers and trimers**		
**Eumelanin**	22 DHI H2Q	218	236	203
	24 DHI H2Q	220	234	208
	27 DHI H2Q	228	242	214
	44 DHICA H2Q	230	241	226
	47 DHICA H2Q	229	242	218
	DHI_trimer_1_H2Q	211	231	195
	DHICA_trimer_1_H2Q	232	249	217
**Pheomelanin**	cyclo	284	293	269
	dimer	280	291	270
	BT-TIQ	207	267	191
	tricochromes_1	101	80	95
	tricochromes_2	77	75	71
		**Chemical degradation products**		
**Eumelanin**	PDCA	282	286	273

	PTCA	283	287	274
	PTeCA	293	304	278
**Pheomelanin**	TDCA	260	265	245
	TTCA	251	256	238
	3-AHP	314	313	301
	4-AHP	313	320	301

Table S7. Benchmark of vertical $T_1 \rightarrow S_0$ (in kJ/mol) electronic transitions for selected eumelanin and pheomelanin structures.

		**$T_1 \rightarrow S_0$ (kJ/mol)**					
**System**		LC-BLYP	B3LYP	CAM-B3LYP	M06-2X	r2scan-3c	B2PLYP
**Eumelanin monomers**	DHI H2Q	346	324	339	360	314	341
	DHI IQ	107	84	101	111	74	100
	DHICA H2Q	300	268	289	310	256	290
	DHICA IQ	104	77	96	112	65	93
**Pheomelanin monomers**	5 BT	314	282	306	322	267	305
	5 ODHBT	359	333	353	371	321	X
	5 BZ	352	326	345	367	312	347
**Eumelanin dimers**	22 DHI H2Q	286	244	272	287	228	267
	22 DHI IQ	100	66	90	100	48	84
	44 DHICA H2Q	292	258	282	301	251	280
	44 DHICA IQ	95	65	88	95	46	83
**Pheomelanin dimers**	Dimer BT	348	333	346	362	316	X
	tricochromes_2	181	133	168	180	120	X
**Sorbic alcohol**		327	263	317	344	250	319
